# Revolutionizing Nanovaccines: A New Era of Immunization

**DOI:** 10.3390/vaccines13020126

**Published:** 2025-01-27

**Authors:** Mohammed Saleh, Ahmed El-Moghazy, Adel H. Elgohary, WesamEldin I. A. Saber, Yosra A. Helmy

**Affiliations:** 1 Department of Veterinary Science, Martin-Gatton College of Agriculture, Food, and Environment, University of Kentucky, Lexington, KY 40546, USA; 2 Department of Microbiology and Plant Pathology, University of California, Riverside, CA 92521, USA; 3 Department of Hygiene and Zoonoses, Faculty of Veterinary Medicine, Mansoura University, Mansoura 35516, Egypt; 4 Microbial Activity Unit, Department of Microbiology, Soils, Water and Environment Research Institute, Agricultural Research Center, Giza 12619, Egypt

**Keywords:** nanoparticles (NPs), nanoparticle-based vaccines, preclinical and clinical trials, regulatory challenges, future innovations, food safety

## Abstract

Infectious diseases continue to pose a significant global health threat. To combat these challenges, innovative vaccine technologies are urgently needed. Nanoparticles (NPs) have unique properties and have emerged as a promising platform for developing next-generation vaccines. Nanoparticles are revolutionizing the field of vaccine development, offering a new era of immunization. They allow the creation of more effective, stable, and easily deliverable vaccines. Various types of NPs, including lipid, polymeric, metal, and virus-like particles, can be employed to encapsulate and deliver vaccine components, such as mRNA or protein antigens. These NPs protect antigens from degradation, target them to specific immune cells, and enhance antigen presentation, leading to robust and durable immune responses. Additionally, NPs can simultaneously deliver multiple vaccine components, including antigens, and adjuvants, in a single formulation, simplifying vaccine production and administration. Nanovaccines offer a promising approach to combat food- and water-borne bacterial diseases, surpassing traditional formulations. Further research is needed to address the global burden of these infections. This review highlights the potential of NPs to revolutionize vaccine platforms. We explore their mechanisms of action, current applications, and emerging trends. The review discusses the limitations of nanovaccines, innovative solutions and the potential role of artificial intelligence in developing more effective and accessible nanovaccines to combat infectious diseases.

## 1. Introduction

The world is currently grappling with a series of critical public health challenges, including the emergence of multidrug-resistant bacteria, rapidly mutating viruses, increasing populations of superbugs, anthelmintic-resistant parasites, zoonotic pathogens, and secondary tumors. These challenges, compounded by the recent COVID-19 pandemic, pose significant threats to human health and livestock [1]. These epidemic and pandemic outbreaks have far-reaching consequences, contributing to global mortality rates and hindering socioeconomic development. Additionally, they necessitate robust biodefense strategies to protect populations from future health crises [2,3,4,5]. The overuse and misuse of antibiotics and other drugs have significantly contributed to the rise of antimicrobial resistance, a major global health crisis [6,7]. Additionally, climate change and environmental pollution have accelerated the emergence, transmission, and spread of infectious diseases, further exacerbating the challenges faced by public health systems worldwide [8,9]. Global cooperation has highlighted the need to tackle these rising resistance and infection levels. The One Health Trust has declared that vaccination is considered an integral strategy to combat antimicrobial resistance, slow the spread of superbugs, and lower the overall burden of infection [10,11,12].

The immune system is highly sophisticated and is considered the first line of defense against foreign infectious and non-infectious signals. The innate immune cells are composed of a variety of circulating cells, such as natural killer cells, granulocytes (neutrophils, basophils, eosinophils, and mast cells), and antigen-presenting cells (macrophages and dendritic cells). The immune system is responsible for the processes driving the uptake and ingestion of phagocytosed particles as well as the process associated with breaking them down [13]. The five pattern-recognition receptors that can recognize pathogens play important roles in the initiation and activation of the immune response [13,14]. The intended output of vaccination is to stimulate the innate and adaptive immune systems to induce B- or T-cell responses and provide prolonged protection [15]. When a vaccine introduces an antigen into the body, B-lymphocytes produce antibodies that fight infection, while T-cells recognize and kill cells infected with a virus or other foreign cells, thus controlling the infection transmission and spread [16].

Recently, nanotechnology has provided significant advancements in the medical sector, including the production of nanovaccines, nanotherapeutics, diagnostics, and the improvement of drug screening and gene delivery approaches [17,18,19]. Nanovaccine platforms represent a transformative avenue for vaccine delivery, offering potential solutions to challenges in traditional vaccine development [20]. These platforms use nanoparticles (NPs), such as lipids, polymers, proteins, etc. to improve the delivery of antigens to target cells, resulting in broader and more specific protection against cancers, infectious diseases, inflammatory diseases, atopic diseases, autoimmune diseases, and others [20]. Nanoparticles can be designed to increase the stability and immunogenicity of antigens. They not only serve as antigens carriers but can also function as antigens themselves [21]. NPs can efficiently target vaccine molecules to the desired cell and its receptors, thereby minimizing side effects [22]. Additionally, they have been found to facilitate antigen distribution to APCs and protect antigens and adjuvants from proteolytic and enzymatic degradation [23]. Nanovaccines can evoke both humoral and cell-mediated immune responses [22]. Nanovaccines can persist for a longer time without degradation or alteration, thereby providing enough opportunity for antigen presentation and dendritic cell (DC)-mediated antigen uptake, resulting in mature DC–T-cell interaction, which is necessary to influence the release of pro- and anti-inflammatory cytokines that promote cell-mediated immunity [24]. The NP-mediated vaccine can enhance B-cell antibody production by activating lymphocytes and monocytes. Furthermore, they can enable a targeted release of cargo into draining lymph nodes after passing through the lymphatic drainage system, generating a potent, long-lasting, and antigen-specific immune response [25,26]. These characteristics suggest that NPs might be crucial to vaccines as immune cell stimulators.

In this review, we highlight the transformative impact of NPs on vaccine platforms, focusing on their ability to enhance antigen delivery, improve immune system activation, and address the limitations of traditional vaccines. Specifically, we explore the types of NPs utilized, their mechanisms of action, current applications in vaccine development, and the potential they hold for creating more effective, stable, and accessible immunization strategies in the future. Additionally, we discuss the challenges and opportunities that lie ahead in the development and deployment of nanoparticle-based vaccines.

## 2. Conventional vs. Nanoparticle-Based Vaccines

### 2.1. Conventional Vaccines

**Figure 1:**
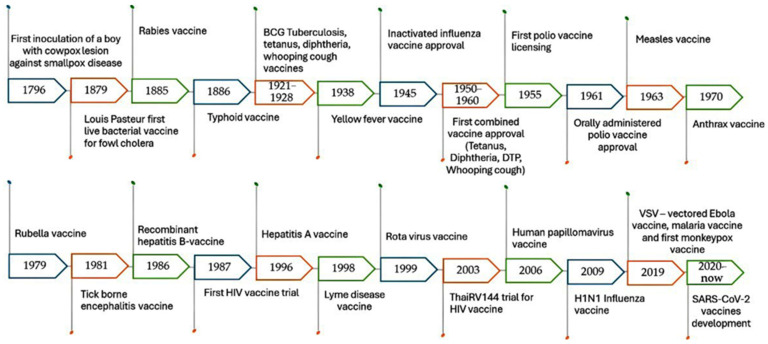
A brief history of conventional vaccine development.

Conventional vaccines are considered one of the greatest advances in medical history against various diseases. They were discovered in the 1700s when Edward Jenner, a British physician, demonstrated that infection with mild cowpox virus can confer immunity against smallpox virus. This discovery led to the development of the smallpox vaccine, which was the first vaccine to be developed against a contagious disease. Consequently, scientists and vaccinologists began occasional and inconclusive experiments for immunization against specific pathogens, as illustrated in Figure 1 [27,28,29,30,31]. The traditional vaccine development pipeline consists of exploratory research, pre-clinical studies, and clinical trials (Phases I, II, and III), followed by regulatory evaluation and approval. However, there are many types of conventional vaccines, which are classified based on their mechanisms of action.

#### 2.1.1. Inactivated Vaccines

This type contains killed pathogens such as influenza, coryza, typhoid, cholera, mycoplasma, hepatitis A, rabies, *Brucella abortus*, *Mannheimia haemolytica*, and *Erysipelothrix rhusipathiae* vaccines [19,32,33]. These vaccines do not offer as much protection as live vaccines do, so booster shots are often required to maintain immunity.

#### 2.1.2. Live Vaccines

Live vaccines contain attenuated forms of pathogens such as measles, rotavirus, avian infectious bronchitis, Newcastle disease, bovine viral diarrhea, canine distemper, and fowl pox [34]. The immune system responds to this type of vaccine by producing strong and long-lasting protection. However, they may not be suitable for hosts with weakened immune systems, due to the possibility of reversion to virulence, interference with maternal immunity, and tissue damage, which may result in pathological disorders or secondary bacterial infection.

#### 2.1.3. Subunit Vaccines

Subunit, recombinant, polysaccharide, and conjugate vaccines utilize specific microbial components, such as proteins, sugars, or capsules, to induce a targeted immune response. Examples include vaccines for hepatitis B, human papillomavirus, and shingles. While these vaccines offer several advantages, including suitability for immunocompromised individuals, they may require booster doses to maintain long-lasting immunity. Additionally, the absence of pathogen-associated molecular patterns in subunit vaccines can limit their ability to stimulate a robust immune response [35,36].

#### 2.1.4. Toxoid Vaccines

Toxoid vaccines, such as tetanus and diphtheria vaccines [37], contain attenuated bacterial toxins, and have a more effective anti-toxin immune response [38,39]. These conventional vaccines have different administration routes, including intramuscular, subcutaneous, ocular, oral, and intranasal routes [13].

Traditional vaccines offer several advantages, including safety, proven efficacy in disease prevention, and the establishment of strong and long-lasting immunity [40,41]. However, their main limitations include a lack of genetic stability, the potential of mutations in live vaccines, and a limited ability to induce a robust immune response for long-lasting immunity for inactivated vaccines [42,43,44]. This has led to the emergence of nanovaccines as a next-generation approach.

### 2.2. Nanoparticle-Based Vaccines

NPs can be defined as ultrafine particles with definite dimensions that exhibit unique physicochemical characteristics, supporting their application in various biomedical and food quality fields [45,46,47]. They have emerged as promising vectors in vaccine technology development and are considered a promising alternative to conventional vaccines (Table 1) [48,49]. Studies have revealed that the physicochemical properties of NPs can influence the immune response in terms of factors such as particle size, hydrophilicity/hydrophobicity ratio, the number of antigens associated with each particle, and the ability of an antigen to raise a B- or T-cell response. Smaller-sized particles can pass biological barriers and epithelia more easily when compared with particles ranging from 20 to 50 nm in diameter [46]. The size of nanoparticle-based vaccines is similar, to some extent, to the size of intracellular components, making it easy for them to enter cells and induce robust and long-lasting immune responses against intracellular pathogens [50]. Spherical NPs induce the most effective immune responses when compared to other nanoparticle shapes [51]. Hydrophobic NPs are more efficiently taken up by specific immune cells and potentially enhance the immune response when compared with hydrophilic NPs that produce a weakened immune response [52,53]. Negatively charged NPs interact more efficiently with macrophages, while positively charged NPs interact more efficiently with dendritic cells [54]. NPs can be composed of polymers, lipids, proteins, emulsions, nano-beads, inorganic nanomaterials, and virus-like particles and have been approved for both animal and human use to prevent infectious and non-infectious diseases [55].

The major types of NPs used for vaccinology against infectious and non-infectious diseases are illustrated in Table 2. They exhibit a wide range of properties that make them valuable humoral and cellular immune stimulators [13]. They can enhance immune responses by effectively presenting or transporting antigens and allowing precise targeting of immune cells [56,57]. They protect antigens from degradation before reaching their target, thus improving vaccine efficacy and shelf life [58]. Some NPs can act as adjuvants themselves, thus stimulating the immune response without the need for additional substances to the vaccine formula [59]. NPs can be designed to control antigen release, extending immune response duration [60]. Additionally, NPs can be tailored for specific pathogens, making them versatile tools for preventing and controlling infectious diseases [49,61,62,63,64]. The nanoparticle-based vaccines have different administration routes, including ocular, oral, nasal, sublingual, topical, intradermal, intramuscular, and subcutaneous routes. The subcutaneous route is considered the most advantageous route in stimulating the immune response, as vaccines are injected directly into a site that is rich in immune cells, resulting in the production of antibodies at high concentrations [65].

**Figure 2:**
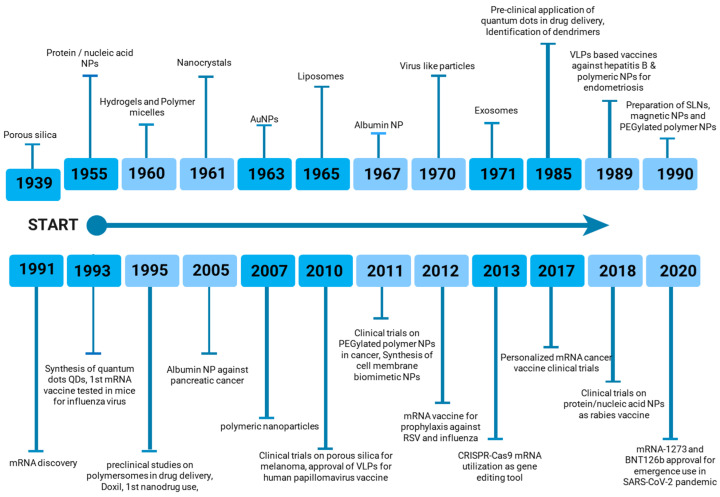
Timeline of nanoparticle-based vaccine development.

The increasing diversity of nanoparticle-based vaccines has prompted ongoing research toward the optimization of delivery methods and the maximization of immune responses against specific antigens. As depicted in Figure 2, nanoparticle-based vaccines have gained significant prominence in the pharmaceutical and biological industries over the past century. The continued development and implementation of nanoparticle-based vaccines hold immense potential to address a wide range of infectious diseases (viral, bacterial, fungal) and non-infectious diseases (autoimmune diseases, cancer) that pose substantial global health challenges [48,49].

Despite significant advancements in nanovaccine development for combating infectious diseases, inflammatory conditions, allergic diseases, and cancers, several challenges persist. Firstly, ensuring consistent size, shape, and composition of NPs remains a complex task, as these properties are crucial for achieving optimal pharmacological, biological, and physicochemical characteristics [149,150]. Secondly, unintended immune reactions or toxicity can arise from nanoparticle accumulation in tissues and organs [20]. Thirdly, stability and shelf-life, under various environmental conditions, pose challenges, particularly for lipid NPs that are sensitive to temperature fluctuations [151,152]. Fourthly, regulatory hurdles, production costs, and safe waste disposal are additional considerations [153]. Lastly, mRNA-based nanovaccines face specific challenges, including the selection of appropriate antigens, the synthesis and purification of mRNA, and ensuring efficient encapsulation within lipid NPs [154].

## 3. Types of NPs Used in Vaccines

**Figure 3:**
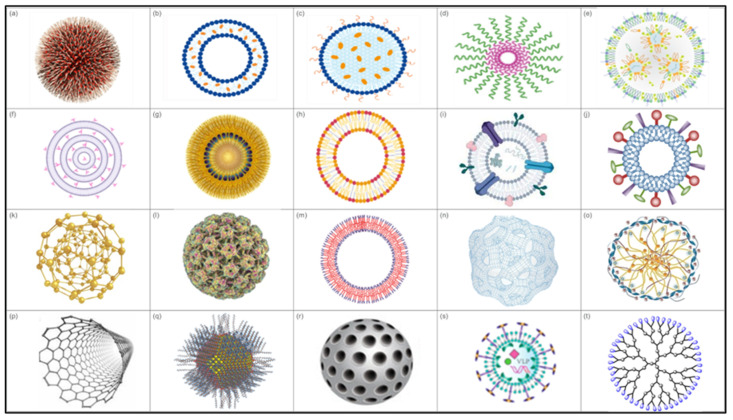
Types of NPs. (**a**) Polymeric nanoparticle, (**b**) liposome, (**c**) solid lipid NP, (**d**) polymeric micelle, (**e**) lipid NP, (**f**) multilamellar lipid vesicles, (**g**) lipid nanoemulsion, (**h**) lipoprotein, (**i**) exosomes, (**j**) virosomes, (**k**) gold NP, (**l**) protein-based NP, (**m**) polymersomes, (**n**) polymeric nanosphere, (**o**) chitosan NP, (**p**) carbon nanotubes, (**q**) quantum dots, (**r**) mesorphous silica NP, (**s**) virus-like particle (VLP), and (**t**) dendrimer.

Nanoparticles exhibit specific characteristics that render them more effective for enhancing the immune system and delivering antigens in a controlled and targeted manner [57,155,156]. Several types of NPs are being explored for vaccine development, including lipid-based NPs, polymeric NPs, self-assembled protein or peptide NPs, biomimetic NPs, and inorganic NPs (Figure 3).

### 3.1. Lipid-Based NPs

Lipid-based nanoparticles (LNPs) are also known as lipid-based nanocarriers. They are spherical NPs that use ionizable lipids as their main structural component. These NPs can be categorized based on their structure and composition. In particular, LNPs offer advantages such as biodegradability, biocompatibility, and safety, which are ideal characteristics for a vaccine delivery system [157]. LPNs can facilitate the delivery of mRNA and protect it from rapid degradation by RNases [68]. Additionally, LNPs can interact with the immune system, enhancing cellular and humoral immunity. They have been pivotal in the development of vaccines and other nanomedicines [68,158]. Furthermore, there are many examples of lipid-based NP vaccines that have been widely used in preclinical and clinical studies (Table 3).

#### 3.1.1. Interbilayer-Crosslinked Multilamellar Vesicles (ICMVs)

ICMVs have an aqueous core surrounded by multilayers of lipids. Their robust lipid wall protects the particles from serum-mediated degradation, enabling stable delivery of vaccine components to lymphoid tissues and thus generating potent antibody responses. ICMVs can serve as a delivery system for both hydrophilic and hydrophobic antigens and adjuvants, showing promise as a vaccination strategy against cancers and intracellular pathogens [181]. Studies have demonstrated that ICMVs with both surface-conjugated and encapsulated malarial antigens offer superior vaccination protection compared to soluble antigens due to the prolonged persistence of antigens in the draining lymph nodes [182]. Despite these values, ICMVs have some disadvantages, such as the complexity of production, the risk of triggering an unintended immune response, the unpredictable release of encapsulated substances from ICMVs, and the fact that techniques used to produce ICMVs may not be easily scaled up for industrial production, limiting their widespread application [181].

#### 3.1.2. Lipoproteins

Lipoproteins are complexes of proteins and lipids. They have been reported to transfer and target various lipids, hormones, proteins, vitamins, and endogenous microRNA to recipient cells [183]. They have a hydrophobic core and a hydrophilic surface, that can facilitate the delivery of a broad range of molecules and act as vaccine-delivery carriers [184]. Recombinant lipoproteins with built-in immune stimulators have been established for novel subunit vaccine development. This core platform technology has demonstrated safety in meningococcal group B subunit vaccine, dengue subunit vaccine, novel subunit vaccine against *Clostridium difficile* and HPV-based immunotherapeutic vaccines in animal model studies [185]. The main disadvantage of lipoprotein NPs is the stability issues as they can be unstable in the bloodstream, leading to premature release of their cargo [186]. Additionally, the manufacturing process can be complex and costly, requiring precise control over conditions to ensure consistency and efficacy [187]. They are potentially toxic and some lipoprotein NPs can trigger immune responses and cause adverse reactions [188,189].

#### 3.1.3. Liposomes

Liposomes are composed of phospholipid bilayers and range in size from 50 to 500 nm in diameter. They are amphiphilic in nature, with hydrophobic surfaces and a hydrophilic core. Liposomes have gained significant attention in delivering immunostimulatory molecules, drugs, DNA, and RNA [190]. They are highly versatile, biocompatible, safe, and capable of creating different structures by modulating components of the lipid matrix. They act as carriers for vaccines and facilitate the targeted delivery of viral proteins, triggering antibody production. Liposome carriers influence antigen internalization by immunocompetent cells and immune response induction [191]. They can act as adjuvants employed in nanovaccines formulation, so prolong stability and trigger antigen-presenting cells to initiate an immune response. The impact of liposomes on antigen-presenting cells depends on many factors, such as size, charge, and lipid composition [192]. Immunostimulant components (ligands of pathogen-associated molecular pattern receptors) can be added to liposomal vaccine complexes to modulate the strength and type of immune response [192]. Furthermore, liposomes can induce immunologic reactions to antigens adsorbed on the surface or encapsulated internally [193]. Several studies have shown that liposome-based vaccines have been widely used in preclinical and clinical studies [194]. However, liposomes have some drawbacks as they can be prone to leakage and fusion with the encapsulated drug, which can reduce their effectiveness [195]. Moreover, the manufacturing process for liposomes is often expensive and complex and they may need to be stored at specific temperatures to maintain stability. They also have been reported to have a short half-life in the bloodstream, which can limit their effectiveness [196].

#### 3.1.4. Solid Lipid Nanoparticles (SLNs)

SLNs are particulate carrier systems that are composed of a solid lipid core and a cationic lipid surface [197]. These SLNs can be used to bind DNA, forming an SLN/DNA complex or lipoplex, which shows promise as a potential vaccine [198]. SLNs offer several advantages, including the ability to encapsulate both hydrophilic and hydrophobic drugs, protecting them from degradation in the body and destruction by enzymes. These properties make SLNs suitable for delivering various active ingredients, including vaccines, thereby enhancing therapeutic efficacy [199,200]. SLNs have some limitations, as some biomolecules can be expelled from these NPs during storage. Additionally, the high water content in lipid dispersions can affect stability and drug concentration. SLNs also have a limited ability to deliver drugs through the skin [201].

#### 3.1.5. Exosomes

Exosomes are small vesicles composed of lipophilic bilayers ranging from 50 to 100 nm in size and are used in the formation of nanovaccines. They can cross biological barriers, including the blood-brain barrier, and deliver biological and therapeutic antigens to specific tissues [202,203]. Exosomes are biocompatible and biodegradable particles, so they are safe for clinical use. Additionally, they are immunogenic and can induce the immune response to the vaccine antigen [202]. It has been reported that exosome-based vaccines can improve the therapeutic index against cancer and immunosuppressive conditions [204]. Ongoing research is evaluating the immune response of exosome-based vaccines against Ebola virus, influenza and COVID-19 [107,205].

#### 3.1.6. Virosomes

Virosomes are structures composed of viral proteins and liposomes, ranging from 20 to 50 nm in diameter and with hydrophobic surfaces that facilitate antigen loading. The viral protein components could be glycoproteins of different viruses, such as herpes and influenza viruses [206]. Inflexal^®^V, an approved FDA nanovaccine, has been used as a subunit influenza vaccine that uses virosomes as carriers to enhance the immune responses against influenza H1N1, H3N2, and B [207].

#### 3.1.7. Emulsions

Emulsions, typically consisting of oil-in-water dispersions, are widely used as delivery systems for vaccines. By incorporating antigens, surfactants, and adjuvants, these emulsions can enhance the immune response to the vaccine. Emulsion-based vaccines have shown significant potential for combating various infectious diseases, including human papillomavirus, hepatitis B virus, and influenza viruses. One notable example of an emulsion-based vaccine adjuvant is MF59, which is licensed for use in certain influenza vaccines [208,209].

### 3.2. Polymeric NPs

Polymeric nanoparticles (PNPs) have served as versatile carriers for active compounds, either absorbed onto their polymeric core or entrapped within the structure itself. PNPs can be synthetics that are chemically synthesized in the laboratory or natural, originating from biological sources such as polysaccharides, proteins, and lipids. Natural PNPs have more advantages over synthetic PNPs in terms of biodegradability, biocompatibility, and low toxicity [210]. There are different PNP design structures such as nanospheres, micelles, and polymerases. Poly (lactic-co-glycolic acid) (PLGA)-based NPs, chitosan, hyaluronic acid, silk fibroin, alginate, polyethyleneimine, polypropylene sulfide, and polystyrene NPs are the major types of polymeric NPs that are commonly used in vaccine adjuvant delivery systems [211,212]. The production of polymeric NPs generally follows two main strategies, the polymerization of monomers or the dispersion of preformed polymers [213].

PNPs can encapsulate multiple adjuvant materials or antigens within the polymer matrix or adsorb it onto the surface, this can protect these biomedical molecules from degradation and enhance their stability. The release of biomedical molecules can be controlled through diffusion, degradation of the polymer, or a combination of both, this allows sustained and targeted delivery. PNPs can be modified with ligands, antibodies, or other molecules that target specific cells or tissues to ensure that the biomedical molecules are delivered precisely to the desired site, reducing side effects and improving efficacy. Some PNPs are designed to release their payload in response to specific stimuli such as pH, temperature or enzymes present in the target tissue [214]. In addition, PNPs are often taken up by cells through endocytosis, a process where the cell membrane engulfs the NPs. Once inside the cell, NPs can release their biomedical molecules payload in tumors and inflamed tissues, meaning that PNP-based vaccines show significant potential as anti-tumor agents [215]. PNPs can also protect labile antigenic particles from enzymatic degradation in vivo [216,217]. Other biodegradable polymers, such as poly-lactic acid, polyethylene oxide, polyethylene glycol, and poly-ε-caprolactone are also used as adjuvants to boost vaccine immunogenicity [218,219]. Polymeric micelles are self-assembled amphiphilic structures that can efficiently deliver viral proteins. The main advantage of micelle NPs is their ability to present antigens in native-like conformations; however, their poor stability in vivo is a major drawback [220,221]. Chitosan NPs have also been used to improve pulmonary immunity against TB by increasing immune responses at mucosal sites [222]. Overall, the main advantages of PNPs include precise control of particle characteristics, payload flexibility, and ease of surface modification. However, there are potential drawbacks, including the possibility of aggregation and toxicity [223], the complexity of their production, their stability under certain conditions, and the fact that some PNPs could be cytotoxic [156,224,225]. There are different applications of PNPs, either in cancer therapy for the targeted delivery of chemotherapeutic agents or in gene therapy applications through the delivery of genetic materials such as DNA or RNA [215,226]. They have also been investigated against malaria, HIV, COVID-19, and tuberculosis diseases [227]. The main examples of polymeric-based nanovaccines that have been widely used in pre-clinical and clinical trials include NVX-CoV2373, a vaccine produced by Novavax which delivers the spike protein from the original Wuhan strain of SARS-CoV-2. The spike proteins self-assemble into 3D structures called “trimers,” which are purified and mixed with an immune-stimulating substance (adjuvant) to form NPs [86]. Clinical trials in adults given two shots of this vaccine 21 days apart demonstrated about 90% efficacy in preventing infection after seven days from the second shot [85,228]. Another example of a PNP-based vaccine is GBP510, a candidate COVID-19 vaccine that has recently undergone a combined Phase 1/2 trial. GBP510 leverages nanoparticle bioengineering technology to stimulate immune responses against COVID-19 [229,230]. Additionally, the TAK-101 vaccine, which incorporates gliadin encapsulated within PLGA nanoparticles (NPs), has been developed for celiac disease and simian immunodeficiency virus (SIV). These PLGA NPs are co-formulated with TLR4 and TLR7/8 agonists, and have demonstrated the ability to induce a prolonged, robust, and high level of protection against SIV in macaque models [231].

### 3.3. Inorganic NPs

Inorganic NPs are biocompatible, non-toxic, hydrophilic and highly stable particles, presenting significant advantages over their organic counterparts. They can be classified into solids (hydroxyapatite and calcium phosphate) or porous (porous silicon and mesoporous silica) particles. Inorganic NPs exhibit unique properties that hold great promise for enhancing immune responses, improving antigen delivery efficiency, and enabling applications in tumor imaging, drug delivery, vaccine development, and infectious disease prevention. These NPs offer advantages, such as enhanced stability, controlled release kinetics, and targeted delivery, which can significantly improve the efficacy of therapeutic interventions [232]. Gold nanoparticles (AuNPs), carbon nanotubes, quantum dots, and silica NPs are among the inorganic NPs.

AuNPs exhibit unique physical, chemical, and optical properties that differ significantly from bulk gold, making them valuable for a wide range of high-technological applications, such as organic photovoltaics, drug delivery, vaccine development, and catalysis [233,234]. AuNPs are commonly referred to as colloidal gold when dispersed in water. Their most pronounced properties are the intense absorbance and scattering of incident light at their surface plasmon resonance wavelength [235]. AuNPs have garnered attention in vaccine development due to their ability to enhance immune response, making them potential adjuvants for vaccines. AuNPs can be functionalized with drugs to enhance targeted delivery and reduce side effects [236,237,238]. Additionally, they provide stability to vaccine formulations during storage [239,240]. Several studies have demonstrated the potential of AuNPs in vaccine development; for example, conjugated antigenic peptides from the SARS-CoV-2 spike protein to AuNPs have shown no inherent toxicity and have been shown to induce significant IgG antibody levels in tested mice [75]. Another example is the increase in the efficacy of the influenza vaccine by over 25% in specific strains through chiral AuNPs [241]. Moreover, AuNPs can attach to and disrupt the cell membranes of bacteria and viruses, leading to cell lysis and death. Additionally, they can induce the production of reactive oxygen species, which cause oxidative stress and damage DNA, proteins, lipids, and other cellular components. They can also trigger apoptosis (programmed cell death) by activating various cellular pathways and can disrupt the metabolic processes of microbial cells through the inhibition of the activity of essential enzymes. AuNPs can induce cellular and humoral immune responses against enterohemorrhagic *E. coli* through increasing antigen uptake and processing [242]. Additionally, they can be loaded with platinum on copper nanosheets, and can promote the glucose oxidation process in tumor tissues, so are considered an optional therapeutic for cancer [243].

The primary limitations associated with AuNPs include (a) potential cytotoxicity at high concentrations, despite their inherent biocompatibility; (b) the requirement for specialized, often complex, and costly techniques for their synthesis and functionalization [244]; (c) their susceptibility to aggregation over time, which can compromise their stability and functionality [245]; and (d) the possibility that they might induce adverse effects like inflammation and immune responses [246].

Silica nanoparticles (SiO_2_ NPs), also known as nanosilica are considered another type of inorganic NP. They are typically amorphous substances with a spherical morphology, although they can be synthesized in various shapes and sizes [247]. Their surfaces can be easily modified for specific purposes, making them suitable nanocarriers for drug delivery due to their high surface area and their ability to adsorb active components [248]. SiO_2_ NPs possess several advantages including the following: (a) they exhibit low toxicity and are considered safe for biomedical applications; (b) they are thermally stable and can withstand high temperatures without significant degradation; (c) silica is abundant and cost-effective; and (d) their surfaces can be modified and functionalized for specific applications and have gained attention as potential adjuvants for vaccine development. For instance, mesoporous silica nanoparticles (MSNs) are nanomaterials with tunable particle size, high porosity, and versatile surface chemistry. They are commonly used for controlled drug release and delivery due to their low toxicity and biocompatibility [249]. Traditional MSNs have small pores (typically 2–6 nm), which limit their utility for immunotherapy since many antigens and immunostimulatory factors are macromolecules (e.g., proteins, nucleic acids). Recently, MSNs have been developed with extra-large pores (20–30 nm) to incorporate antigens for more effective cancer vaccines. Large-pore MSNs can efficiently load macromolecules, making them suitable for immunotherapy [77]. They target dendritic cells in lymph nodes, delivering tumor antigens and activating DCs, which leads to a cytotoxic T lymphocyte (CTL) antitumor response. Overall, silica-based nanomaterials are biodegradable, biocompatible, and easily conjugated with antigens and adjuvants [250]. They exert immunostimulatory effects, enhancing the immunogenicity of subunit vaccines and offering promise in cancer immunotherapy [78]. There are ongoing clinical trials for various cancers, including ovarian cancer, prostate cancer, and glioblastoma [251]. The primary drawbacks of SiO_2_ NPs include the complexity of their synthesis processes and the potential long-term environmental impacts and health risks associated with exposure. These risks may include inflammation, oxidative stress, and cytotoxicity [252,253,254].

### 3.4. Virus-like Particles (VLPs)

VLPs are non-infectious, non-replicating viral structural proteins that lack viral genetic material. They mimic the structure of real viruses and do not cause disease. VLPs are safe and effective immune stimulators [255], highly immunogenic, and capable of inducing both cellular and humoral immune responses. They serve as an alternative platform for developing vaccines against infectious diseases, including hepatitis B, malaria, and SARS-CoV-2. VLP-based vaccines have the advantages of efficacy, safety, stability, and diversity [82,83]. Most licensed VLP-based vaccines use classic aluminum adjuvants, which enhance antibody responses through complex cellular reactions and local antigen trapping at the injection site, ensuring slow release and prolonged stimulation of the immune system [256]. The main drawbacks of VLPs include the following: (1) they can be less stable compared to other NPs; (2) they degrade or lose their structural integrity under certain conditions, which can affect their efficacy; (3) the removal of impurities and the achievement of high purity levels of VLPs is a labor-intensive and time-consuming process; (4) their sensitivity to environmental conditions such as temperature and pH can limit their storage and handling options; and (5) VLPs produced in bacterial systems may contain endotoxins, which can cause severe immune reactions [82,255,257,258].

There are numerous examples of VLP-based vaccines in various stages of clinical and pre-clinical development. For instance, VLPs derived from the hepatitis B virus and human papillomavirus (HPV) have been successfully commercialized. Recently, VLP-based vaccines targeting emerging pathogens such as SARS-CoV-2 have shown significant promise. These include VLPs displaying the SARS-CoV-2 spike protein, which have entered clinical trials [259,260]. Additionally, VLP-based vaccines are being explored for other diseases such as influenza, HIV, and malaria. By mimicking the structure of native viruses, VLPs can induce strong immune responses, making them a versatile platform for vaccine development [261,262,263,264].

### 3.5. Self-Assembling Peptide/Protein Nanovaccines

Self-assembling peptides and proteins can form diverse nanostructures, including nanofibers, NPs, and hydrogels. These structures offer unique advantages for vaccine delivery, such as controlled antigen release, enhanced immunogenicity, and targeted delivery to immune cells [265]. Recent advancements in peptide and protein engineering have enabled the development of self-assembling nanovaccines for various diseases. For example, ferritin-based NPs have been explored as a platform for delivering multiple antigens, including those targeting EBV, SARS-CoV-2, and cancer [266,267]. Additionally, peptide-based vaccines have been designed to induce specific immune responses against various pathogens [267,268].

## 4. Mechanism of Action of Nanovaccines

NPs can be functionalized as antigen carriers through encapsulation or surface conjugation. In encapsulation, antigens are enclosed within the NP matrix, providing a controlled release mechanism. In surface conjugation, antigens are attached to the NP surface, facilitating direct interaction with the immune system [59,269]. The mechanism of action of NPs is governed by their structure, size, surface charge, concentration, dosage, uptake, and processing by the immune system cells [270]. The size of NPs enhances tissue penetration and enables immune cell interaction and activation at the infected site [46]. They can protect antigens from premature enzymatic degradation and facilitate a sustained release, thereby ensuring prolonged antigen exposure and enhancing the immune response [55,271]. NPs can mimic the invasion process of pathogens, enhancing the immune response by presenting antigens more naturally and effectively [87]. NPs can also be functionalized with ligands that attach to certain receptors on antigen-presenting cells, including dendritic cells or macrophages. This targeted approach increases the uptake of antigens by the key immune cells. Once inside the APCs, the NPs degrade, releasing the antigens that are processed and presented on the surface of APCs by major histocompatibility complex molecules [272]. Potentially, the immune response to nanovaccines involves antibodies generated for both the NP itself and the incorporated antigen [273].

Adjuvants can be defined as substances added to vaccines to enhance the immune response, improve antigen presentation, activate the immune cells, and prolong antigen exposure. NPs can act as effective adjuvants by gradually releasing antigens and being engineered to specifically reach antigen-presenting cells. This targeted delivery optimizes immunization strategies, allowing effective immune activation with lower antigen doses [227,274].

Various types of NPs, including gold, dendrimers, carbon, polymers and liposomes, can induce cytokine and antibody responses. LNPs are particularly effective in encapsulating mRNA and delivering antigens to immune cells, facilitating CD8+ T cell activation. These LNPs have demonstrated success in clinical applications, such as in mRNA vaccines for SARS-CoV-2, where they enable efficient delivery of spike proteins to antigen-presenting cells, thereby triggering a strong immune response [189,275]. Additionally, NPs allow for the delivery of multiple antigens within a single vaccine. For example, a single NP could encapsulate spike proteins from various coronaviruses, potentially offering broader protection against related pathogens [276].

## 5. Nanovaccines for Food Safety

Foodborne and waterborne bacterial pathogens continue to pose significant global health challenges, causing substantial morbidity and mortality worldwide. Recent advances in NP-based vaccines have also emerged to form a promising strategy to combat these pathogens through immunization.

Recent studies have demonstrated significant progress in developing nanovaccines against *Salmonella* spp. Chitosan NPs conjugated with *Salmonella* outer membrane proteins have been shown to induce robust immune responses in broilers, protecting against lethal challenges. The nanoformulation showed superior stability when compared with conventional vaccines and also increased mRNA expression of toll-like receptors [277]. Additionally, oral vaccine administration (*S.* Enteritidis immunogenic outer membrane proteins and flagellin entrapped in mannose chitosan NPs) elicited cross-protective mucosal immune responses against *S.* Typhimurium colonization in broilers [278]

Innovative approaches using gold NPs conjugated with *E. coli* O157:H7 antigens have shown promising results. Recent studies have shown that vaccination with gold NPs conjugated to *E. coli* O157:H7 antigens elicits strong systemic and mucosal immune responses, leading to significant protection against *E. coli* O157:H7 colonization [279]. Furthermore, nanoformulations, combining antibiotics, NPs, and polymeric carriers, can improve drug delivery and efficacy in the treatment of urinary tract infection (UTI) treatment, especially UTIs caused by *E. coli*. These nanoformulations can enhance drug solubility, target specific sites of infection, and control drug release, leading to improved therapeutic outcomes and reduced side effects. These advancements in nanotechnology hold the potential to revolutionize UTI treatment by providing more effective and targeted therapeutic options [280].

A novel lipid nanoparticle (NP)-based vaccine incorporating the cholera toxin B subunit has been developed to combat *V. cholerae*. This formulation has demonstrated enhanced mucosal immunity compared to traditional oral cholera vaccines. Additionally, it has shown improved stability under gastric conditions and increased bioavailability, making it a promising alternative for cholera prevention [281]. Complementary research has shown that nanoparticle-based vaccines combining *V. cholerae* outer membrane vesicles induced long-lasting immunity [282].

Engineered polymer-based NPs incorporating flagellin and outer membrane proteins of *Campylobacter jejuni* have shown a significant reduction in bacterial colonization, achieving protection in poultry models [109]. Additionally, hybrid NPs combining chitosan and alginate have shown enhanced mucosal adherence and improved immune responses against *C. jejuni* [283,284].

Advanced NP formulations targeting *L. monocytogenes* have emerged as potential preventive strategies, where developed NPs conjugated with listeriolysin O have demonstrated enhanced cellular immunity and effective cross-presentation to CD8+ T-cells [285]. In addition, NPs loaded with multiple listeria antigens achieved broad-spectrum protection against different serotypes [285].

Innovative approaches in *Shigella* nanovaccine development have shown significant progress using NPs encapsulating *Shigella* invasion plasmid antigens, demonstrating robust protection against multiple *Shigella* species [286]. Furthermore, success has been reported with pH-responsive NPs that specifically release antigens in the intestinal environment, enhancing the efficiency of immune response induction [287].

## 6. Nanoparticle-Based Vaccines in Veterinary Medicine

The advancements in nanotechnology have revolutionized the field of vaccination and drug delivery, significantly enhancing the diagnosis, prevention, and treatment of both infectious and non-infectious diseases in humans and animals [269,288]. NPs, when used in vaccines, enable targeted delivery, enhance immune response, and potentially offer prolonged immunity, making them particularly advantageous in veterinary medicine applications. By reducing the reliance on antimicrobials, these nanovaccines play an essential role in promoting animal welfare and addressing antimicrobial resistance, a growing concern in veterinary and public health [289,290]. In veterinary medicine, nanoparticle-based vaccines have demonstrated effectiveness in conferring specific and durable immunity against a range of clinically significant bacterial pathogens, including *Salmonella* spp., *E. coli*, *Staphylococcus* spp., *Helicobacter* spp., *Mycobacterium* spp., *Clostridium* spp., and *Pseudomonas* spp. For example, studies have demonstrated that nanoformulations can enhance antigen stability and allow for controlled release, which can improve vaccine efficacy and immune memory [288,289,290]. This technology is also being explored to develop novel vaccine platforms that may improve immune responses against difficult-to-treat pathogens and reduce disease burden in livestock industries [290].

Secreting membrane vesicles are nanostructures that can stimulate T-cell responses against different antibiotic-resistant bacteria such as *S. aureus* and avian pathogenic *E. coli*. These extracellular vesicles produced by resistant bacterial strain surfaces and coated with indocyanine-loaded magnetic silica NPs could significantly reduce surface infection, systemic invasiveness, and complications of *S. aureus* in experimental models [97]. The outer membrane vesicles of avian pathogenic *E. coli* serotype O2 have experimentally promoted strong specific and non-specific protective immune responses against homologous infection with *E. coli* in broilers, which was achieved via the reduction of bacterial loads, reduction of proinflammatory cytokine production and activation of T-cell responses [98].

Carbon nanotubes and poly-anhydrous NPs have been tested against anaplasmosis or inactivated *M. paratuberculosis* in murine experimental models. The carbon nanotubes absorbed and displayed the *A. marginale* MSP1 protein on both their outer and inner surfaces. They then traversed biological membranes without compromising their integrity. In contrast, the polyanhydrides encapsulated whole-cell lysates and *M. paratuberculosis* culture filtrates through nanoprecipitation. [99].

Furthermore, nanovaccines based on the oligo-polysaccharide antigen and poly (lactic-co-glycolic acid) NPs have shown promise against *B. melitensis* through efficient engulfment by macrophages and other phagocytic cells [117]. Polyanhydride NPs encapsulating pathogenic fusion proteins co-adjuvanted with noncanonical CDGs are among the latest innovations that rapidly induce specific protective immune responses against highly virulent *B*. *anthracis* and *Y. pestis* [111]. *F. columnare*, which is a highly contagious bacteria affecting tilapia, can be controlled and suppressed by the immersion of mucoadhesive polymer chitosan-complexed nanovaccine, which modulates the mucosal immune response of tilapia against columnaris disease [103]. Avian pathogenic *E. coli*, which is a highly infectious pathogen impacting poultry production worldwide, has been minimized in chickens by using chitosan-based nanovaccines. These chitosan nanovaccines can encapsulate and load the outer membrane protein and flagellar antigen, resulting in improved vaccine efficacy and protection against avian pathogenic *E. coli* [108].

Additionally, chitosan nanovaccines containing *S. Enteritidis* that are surface-coated with F-protein and O-F antigen have successfully reduced the load of *S*. Enteritidis in chicken and pig intestines by stimulating specific memory B and T cell responses [109,110,291]. Moreover, nanoparticle-based vaccines have been used for the prevention and treatment of different parasitic infections and infestations such as malaria, leishmaniasis, toxoplasmosis, schistosomiasis, anaplasmosis, theileriosis, trypanosomiasis, and coccidiosis. They have also been applied to parasitic vectors like fleas, mosquitoes, and ticks [147,292]. Inoculation of nanovaccines in experimental animals against *T. cruzi* has been developed and optimized for both the protection and control of parasite replication and dissemination [112]. Moreover, new nanovaccine platforms, such as antigen-absorbing silica vesicles, have been evaluated against tick-borne diseases in laboratory models and have shown very promising results [113]. Another novel nano vaccine has shown synergistic efficacy and balanced immune response against *T. parva* [81]. Furthermore, precisely engineered nanovaccines have provided stable, long-lasting immunological and less toxigenic responses against highly pathogenic and mutated viruses, including avian influenza, RSV, and RVF virus [104,147,293,294].

## 7. Current Research on Nanoparticle-Based Vaccines

The use of NPs in vaccines can improve immunogenicity and offer a promising platform for future vaccine development. Researchers are utilizing microfluidic platforms to evaluate the performance of NPs in preclinical studies. These platforms can mimic in vivo conditions, allowing a more accurate assessment of NPs’ efficacy and safety [295]. In addition, membrane-based nanovaccines are under investigation, with several candidates in preclinical and clinical phases for use as effective cancer nanovaccines to inhibit tumor growth and metastasis [296,297,298]. NPs are being explored to improve the delivery and efficacy of cancer treatments. For instance, in lung cancer therapy, NPs can deliver drugs directly to tumor cells, enhancing the precision and effectiveness of the treatment while minimizing side effects [299]. Different types of NPs, such as polymer NPs, liposomes, quantum dots, dendrimers, and gold NPs, are being studied for their unique properties and applications in targeting tumor tissues [299,300,301]. Animal models remain the appropriate tool in demonstrating the efficacy of nanoparticle-based vaccines. Rats, guinea pigs, mice, sheep, canines, rabbits, swine, and non-human primates like African green monkeys and Asian macaques are among the most commonly used animal models. Small animal models offer advantages in handling, housing, and maintenance costs; however, selecting a suitable animal model is crucial for translating experimental findings to clinical applications and overcoming developmental barriers. For example, mice are more resistant to classic TB disease which minimizes their utilization for evaluating TB vaccines. The encapsulation of antigens in suitable animal models using microparticles/NPs enhances the immune responses, resulting in better immunity and broader protection compared with conventional vaccines [302]. Self-assembled peptide nanovaccines (SAPNs) can act as innovative nanovaccines that enhance antigen-presenting cell uptake, with B-cell activation and lymph node trafficking, resulting in a robust immune response and protection against various animal infectious diseases [303]. Additionally, VLPs have shown promise in vaccine development and have effectively protected animal models against SARS-CoV-2 infection [304].

## 8. Hurdles for Nanoparticle-Based Vaccines

Regulatory approval pathways for NPs can be extremely complex due to their unique features and potential applications across various fields, including medicine, cosmetics, and food packaging. The U.S. Food and Drug Administration (FDA) regulates NPs under existing statutory authorities, tailored to the specific product type (e.g., drugs, biologics, devices). For nanomedicine, the FDA requires thorough evaluations of safety, quality, and efficacy and often involves laboratory and clinical studies due to their novel properties. The regulatory bodies perform a risk-benefit analysis tailored to the intended use of the nanoparticle product with continuous monitoring of nanoparticle products post-approval to ensure ongoing safety and effectiveness [305,306,307]. Regarding NP-based vaccines, key considerations include validation methods for antigen selection, formulation stability, antigen release rates, the characterization of carrier pharmacokinetics and biodistribution, and rigorous clinical trials and safety assessments that include Phase I, II, and III trials which assess safety, efficacy, and immunogenicity [308,309,310].

Several critical considerations and challenges pertain to NPs, including safety concerns related to adverse reactions, as NPs may accumulate in tissues and organs causing harm. Some NPs may trigger allergic reactions, inflammations, and oxidative stress and there is potential for NPs to induce DNA damage, which could result in mutations and genotoxicity [311]. Regulatory bodies like the FDA, European Medicines Agency (EMA), and National Medical Products Administration (NMPA) have set guidelines and standards to ensure the safety and efficacy of these nanoparticle-based vaccines. Another challenge is the cold chain requirements of LNPs, as they are sensitive to temperature fluctuations and thus require stringent cold chain maintenance during storage and distribution [152]. Global harmonization is another challenge, ensuring consistent standards and social considerations across different countries. The U.S. FDA has set specific guidelines, including preclinical studies, clinical trials, and post-marketing surveillance to ensure the quality, safety, and efficacy of nanoparticle-based vaccines. The European Medicines Agency (EMA) also has stringent regulations focusing on safety, efficacy, and quality control for the approval of NP-based vaccines. In China, the NMPA focuses on clinical trial data and post-marketing monitoring for the regulation and approval of NP-based vaccines. Furthermore, regarding social considerations, there may be different levels of vaccine hesitancy due to mistrust or due to misinformation from the healthcare system, because some communities may have philosophical or religious objections to certain vaccines, and also because there may be disparities in healthcare access and distribution, particularly in low-income countries. Global organizations, like the World Health Organization (WHO), are working to harmonize vaccine standards and guidelines to ensure consistency and safety across countries [312]. Ongoing efforts to overcome cultural barriers and educate and engage communities about the importance of vaccination and increase acceptance. The complexity of nanoparticle design is another challenge and requires careful manufacturing to achieve an ideal product with the appropriate pharmacological, biological, and physicochemical characteristics [149]. The ability to overcome biological barriers to deliver therapeutics effectively to the target sites is a further challenge. There are additional challenges for mRNA-based nanovaccines, which include selecting the appropriate target antigen, synthesizing, and purifying mRNA, and ensuring effective encapsulation of mRNA within lipid NPs. There are several manufacturing challenges for NPs, including top-down manufacturing approaches for breaking down bulk materials into NPs. These techniques, such as milling, lithography, and laser ablation, can be energy-intensive and may lead to issues like contamination and non-uniform particle sizes [313]. In contrast, bottom-up, such as chemical vapor deposition, sol-gel processes, and self-assembly, offer greater control over particle size and composition [313].

There are also scalability challenges for NPs, including the need for uniformity and to achieve consistent size, shape, and distribution of NPs on a large scale, which can affect the performance and safety of the final product [313]. Even small amounts of impurities can significantly alter the properties of NPs, making it crucial to maintain purity and prevent contamination during large-scale production [314]. Additionally, scaling up nanoparticle production can be expensive due to the need for specialized equipment and materials.

Current research focuses on optimizing the manufacturing processes for nanovaccines to enhance their scalability, effectiveness, and cost-efficiency. mRNA vaccines have gained attention due to their rapid design, potent immune responses, and safety. Additionally, they can be designed within days, allowing fast production and scalability [315]. LNPs acting as effective delivery platforms for mRNA vaccines can enhance immunogenicity, target delivery, and improve stability [274]. Interestingly, another platform, called the Silicon Scalable Lipid NP Generation platform, has been developed with a scalable and efficient solution for LNP production and for addressing the challenges exposed during the COVID-19 pandemic [93].

Different measures can help in achieving high-quality and consistent NPs, which are essential for their reliable performance in various applications. Advanced techniques like electron microscopy (scanning, and transmission), and dynamic light scattering are used to analyze the size, shape, and distribution of NPs. Developing standardized protocols for synthesis and characterization helps in maintaining consistency across different batches. Implementing strict control over the production process, including temperature, pH, and reactant concentrations, ensures reproducibility. Regular testing and validation of NPs against predefined criteria help in identifying and mitigating any deviations. Using automated systems for production and quality control can reduce human errors and improve consistency. Adhering to regulatory standards and guidelines ensures that the NPs meet safety and efficacy requirements. Furthermore, rigorous quality control and adherence to regulatory guidelines are essential for successful nanoparticle-based vaccine safety and efficacy. The US Pharmacopeia has developed analytical methods by which to assess attributes such as identity verification, impurity control, and content measurement for mRNA vaccine and these quality standards contribute to enhanced regulatory confidence [316,317,318]. Preclinical assessment of nanovaccines involves measuring physical, chemical, and stability properties, as well as assessing immunogenicity and toxicity in vitro and in vivo, which are essential to ensure comprehensive characterization throughout vaccine development [319].

## 9. Innovations in NP Development

### 9.1. Nanocages, Dendrimers, and Other Novel Structures

Various innovative structures, including nanocages and dendrimers, have emerged in biomedical research and vaccine development. Nanocages are nanoscale, hollow structures with porous walls. Nanocages offer unique properties, including porosity, high loading capacity, controlled release mechanisms, and customizable surface properties. These characteristics make nanocages highly effective for delivering therapeutic and biological molecules. Additionally, nanocages are not recognized as exogenous particles and can be biologically or chemically engineered. Nanocages can be classified into two groups: organic nanocages (e.g., protein nanocages, DNA nanocages, etc.) and inorganic nanocages (e.g., gold nanocages, silica nanocages, carbon-based nanocages, etc.) [320]. Organic nanocages, particularly protein nanocages derived from endogenous proteins, exhibit minimal interactions with living cells. In contrast, inorganic nanocages offer high antigen-loading capacity, controllable drug loading and release kinetics, flexible surface chemical modification, and in vivo safety. They can also incorporate luminescent, radioactive, or magnetic reporter molecules for tracing purposes.

Dendrimers are highly branched nanosized polymers with a central core and multiple layers of branching units. Their characteristics, such as chemical stability, polyvalency, electrostatic interactions, self-assembling, solubility, and low cytotoxicity, make them suitable candidates for functionalization with antigens, adjuvants, and other nucleic acid-based vaccines [321,322]. This technology facilitates a rapid response to vaccines, with broader efficacy and reduced vaccination frequency. Notably, dendrimer-RNA nanoparticles have demonstrated significant protective effects against lethal pathogens such as H1N1 influenza, Ebola, and *T. gondii*, with a single dose protecting challenged mouse models [94].

Outer membrane vesicles (OMVs) are spherical double-layered nanostructures (20 to 250 nm) derived from the surface of gram-negative bacteria. OMVs do not cause diseases because they mimic the structures found on the bacterial cell surface and cannot be replicated. They have a promising role in the creation of nanoparticle vaccines and can be engineered to display multiple antigens against different bacterial and viral infections and malignant tumors. OMVs can strongly stimulate the innate and adaptive immune responses [323]. However, there remain some challenges that are being addressed, including possible degradation by proteases, protein misfolding, and inefficient translocation of complex antigens [324].

Metal–organic frameworks (MOFs) are examples of NPs that can be used in nanovaccines to enhance the immune response to antigens. MOFs can encapsulate and deliver viral proteins, such as the SARS-CoV-2 spike protein. Once inside the cells, MOFs break down and activate the innate immune system through toll-like receptors that lead to increased production of cytokines and other inflammatory molecules [325,326].

Nanogels show great potential as novel antigen-delivery vehicles. They are sub-micron structures with a network of cross-linked polymer chains. These nanogels can encapsulate biomolecules and target specific immune cells [327,328]. They also can be designed to release antigens in a controlled manner to prolong the immune response. In addition, nanogels can be designed to display multiple antigens to protect against multiple infectious agents [329].

Combinational nanovaccines can combine multiple antigens in a single formulation to induce a broader and stronger immune response. Despite the potential benefits of combinational nanovaccines, several challenges should be considered, as the multiple antigens and adjuvants in the combined nanovaccines can interfere with each other, resulting in adverse effects, while their design can be complex and requiring increased testing and evaluation. The other challenge is regarding combinational nanovaccines’ safety. There are different examples of combinational nanovaccines in the preclinical and clinical phases, including an influenza vaccine that can protect against multiple strains of influenza viruses [330], a tuberculosis vaccine that targets different TB antigens [331], a cancer vaccine that combines multiple cancer antigens [332] and an HIV combined vaccine that targets multiple strains of HIV [333].

Most traditional vaccines are administered through injection, which could be painful for some individuals. Needle-free nano vaccine administration (oral delivery, inhalation delivery, and transdermal delivery) offers several advantages, including simple administration, proven safety, reduced pain, and robust immunogenicity. For oral nanovaccine delivery to be suitable for GIT diseases, such as cholera and rotavirus infections, NPs must be designed to withstand the acidic pH of the stomach and to release the antigen in the lumen of the gut to initiate the immune response. Transdermal nanovaccine delivery is another delivery approach through which nanoparticle-based vaccines can penetrate the skin and reach the underlying tissues’ immune cells. The inhalation delivery of nanovaccine also ensures that nanoparticle-based vaccines can reach the immune cells in the lung and induce the desirable immune response [334,335,336,337,338]. Overall, needle-free nanovaccine administration offer many advantages, including reducing the risk of blood-borne disease transmission; reducing pain, especially in individuals with needle phobia; eliminating the need for specific equipment and trained personnel due to the ease of administration; and enhancing immunogenicity due to direct delivery of the vaccine to the immune cells.

Interestingly, there have been several suggestions regarding the utilization of spores of *Bacillus* spp. as a mucosal vaccine delivery carrier. These spores have the benefits of dormancy and extreme resistance properties. Many antigens have been presented on these spores through recombinant and non-recombinant methods and have exhibited robust antigen-specific immune responses. Additionally, probiotic-based spores have shown promise in enhancing immune responses by presenting antigens on probiotic strains [339,340].

### 9.2. Using NPs with Other Advanced Technologies

Other important advances in gene therapy in which NPs play a crucial role include the advancement of cutting-edge technologies like Clustered Regularly Interspaced Short Palindromic Repeats (CRISPR)/associated protein 9 (Cas9) and mRNA-based therapies. CRISPR/Cas9 technology is renowned for its precise gene-editing capabilities and is considered one of the most promising technologies for genome manipulation. However, CRISPR/Cas9 has low intracellular delivery efficiency, which severely affects its potency and clinical application, meaning that the development of an effective delivery system for CRISPR/Cas9 system is crucial. LNPs and other nanocarriers like liposomes, cationic polymers, and gold NPs, have emerged as efficient carriers for CRISPR/Cas9 components, overcoming challenges such as inadequate cellular entry, off-target effects, and nuclease degradation [341,342]. A notable advancement in tumor targeting involves the use of a multiplexed dendrimer with an LNP [95]. This system involves the co-delivery of Cas9 mRNA, focal adhesion kinase siRNA, and sgRNA (siFAK + CRISPR-LNPs) to enable tumor delivery and enhance gene-editing efficacy. The study demonstrated that gene editing was enhanced and increased by more than 10-fold in tumor spheroids, due to increased cellular uptake and NP penetration mediated by FAK-knockdown in tumor tissues.

### 9.3. Personalized Vaccines

Personalized nanovaccines represent an exciting frontier in vaccine development, tailored to the genetic variations, existing immunity, age, sex, and disease history of individuals. Unlike traditional vaccines, which have a one-size-fits-all approach (standard antigen formulation and dose), personalized nanovaccines are specifically designed for each host’s unique immune system. Researchers have been able to develop personalized vaccines after analyzing biomarkers that can predict the host immune response to vaccines. The main advantage of personalized vaccines lies in their ability to target the individual’s unique immune system, thereby maximizing efficacy and minimizing adverse reactions [343]. However, their widespread adoption faces challenges including the need for biomarkers development, high costs, and logistical barriers [181,276,344]. Recent examples of engineered personalized nanovaccines include personalized flu vaccines and cancer immunotherapy. In cancer immunotherapy, the tailored nanovaccines not only enhance antigen presentation but also effectively modulate immunosuppression within the tumor microenvironment. A notable example is the mRNA-based personalized cancer vaccine mRNA-4157, which targets twenty tumor-associated antigens specifically expressed by cancer cells, demonstrating the potential for both immunostimulatory and antitumor activities [345]. Fluoropolymer-based nanovaccines represent another class of personalized nanovaccines. When used in combination with immune checkpoint blockade therapy, these tailored nanovaccines have exhibited prevention of post-surgical metastasis and tumor recurrence in breast cancer models [96]. Additionally, neoantigen nanovaccines, which target unique tumor-specific antigens arising from mutations in cancer cells, have shown promise as personalized nanovaccines. For example, a study has demonstrated that delivering neoantigen peptides along with a sting agonist through a nanoparticle-based system significantly improved the immune response and survival rates in a melanoma mouse model [346]. Moreover, the influenza virus is characterized by its rapid mutation, requiring traditional influenza vaccines to be updated periodically according to the circulating strains. However, all individuals cannot equally respond and be protected using these influenza vaccines. These challenges could be controlled with the development of personalized influenza vaccines that optimize the immune response of each individual.

## 10. Artificial Intelligence in Nanoparticle-Based Vaccines

Artificial intelligence (AI) has emerged as a transformative force in the development of NP-based vaccines, revolutionizing multiple aspects of the development pipeline from initial design to clinical implementation. The integration of AI technologies, particularly machine learning, and deep learning approaches, has significantly accelerated the vaccine development process while improving efficiency and predictive accuracy [347]. These advanced computational tools have enabled researchers to optimize antigen design, predict immune responses, and enhance the overall efficacy of NP-based vaccine platforms.

In the field of antigen design and NPs optimization, AI algorithms have demonstrated remarkable capabilities in predicting optimal epitope sequences and enhancing antigen stability. These systems can analyze vast datasets of protein structures and immunological responses to identify the most promising vaccine candidates, significantly reducing the time and resources required for traditional experimental approaches [348]. AI-driven platforms have revolutionized the characterization of nanoparticle properties, enabling precise control over particle size, shape, and surface chemistry, which are crucial parameters for vaccine efficacy [349].

The application of machine learning in formulation development has particularly enhanced our ability to optimize nanoparticle composition and predict stability profiles. These AI systems can analyze complex relationships between formulation parameters and vaccine performance, leading to more stable and effective vaccine candidates [350]. Additionally, AI models have proven invaluable in predicting immunological responses and potential adverse reactions, allowing researchers to optimize vaccine formulations before clinical trials begin [351].

Looking toward the future, the integration of multiple AI technologies presents exciting opportunities for nanovaccine development. The combination of big data analytics with sophisticated AI models is expected to enable more personalized vaccine approaches and improve prediction accuracy. However, challenges remain, including the need for larger, more diverse datasets and the validation of AI predictions in biological systems. As these technologies continue to evolve, they are expected to play an increasingly crucial role in developing next-generation nanovaccines that are more effective, safer, and more readily available to global populations.

## 11. Conclusions

Nanoparticle-based vaccines hold immense promise for revolutionizing immunization strategies. These vaccine platforms offer several advantages over traditional approaches, including enhanced immunogenicity, targeted delivery, and sustained antigen release. The ability to incorporate multiple antigens and adjuvants into a single nanoparticle formulation further simplifies vaccine administration and improves efficacy. However, significant challenges remain in translating these promising technologies into practical applications. Overcoming manufacturing hurdles, ensuring long-term stability, and conducting rigorous clinical trials are essential to bringing NP-based vaccines to market. Addressing potential toxicity and immune side effects is crucial for ensuring their safety and efficacy. Researchers can develop highly effective and personalized vaccines by combining nanotechnology with advancements in artificial intelligence and bioengineering. This interdisciplinary approach will facilitate the creation of vaccines capable of combating emerging infectious diseases, reducing the burden of chronic illnesses, and improving global health outcomes.

## Figures and Tables

**Table 1:** Comparison between conventional and nanoparticle-based vaccines.

Criterion	Conventional Vaccines	Nanoparticle-Based Vaccines
Types	Live attenuated, inactivated, subunit and toxoid vaccines	Protein NPs, lipid NPs, polymer-based NPs, inorganic NPs, and virus-like particles
Advantages	Proven track record, strong and long-lasting immunity	Can carry multiple antigens, enhanced stability, targeted delivery, potentially stronger response
Mechanism	Uses attenuated/killed pathogens or pathogen components to stimulate immune response	Delivers antigens in a targeted manner for better recognition and immune response
Immune response	Immunogenicity can vary and often requires adjuvants to elicit a robust response	Designed for a more specific, potent immune response with built-in adjuvants
Antigen delivery	Direct administration, often less targeted	Targeted delivery of specific cells or tissues
Antigen stability	Require strict temperature control and maintenance	Nanocarriers extend the shelf life of antigens and protect them from degradation
Production Complexity	Long-established manufacturing processes	Advanced bioengineering techniques, more complex production
Stability	Generally unstable, requires cold chain storage	Often more stable, some formulations may require less stringent storage conditions
Customization	Limited adaptability to rapidly mutated pathogens	Highly customizable for emerging pathogens
Dosage	Multiple administrations may be required for optimal immunogenicity, depending on the vaccine and target population	Targeted delivery has minimized the administration of multiple doses
Safety	Almost safe with few risks of reversion to virulent form regarding live attenuated vaccines	Potential toxicity of some NPs
Examples	Polio, hepatitis B, IB, NDV, tetanus, fowl pox	Novavax (protein NPs) and COVID-19 mRNA vaccines (using lipid NPs)

**Table 2:** List of nanoparticle-based vaccines used for treating infectious and non-infectious diseases.

Nanoparticle Type	Targeted Infectious Pathogen/Disease	Pathogen Classification	Reference
Lipid-based NPs	SARS-CoV-2, herpes simplex virus, leishmania and *Schistosoma*	Virus/parasite	[66,67,68,69]
Gold NPs (AuNPs)	SARS-CoV-2, Hepatitis C Virus, Foot and Mouth Disease (FMD), *Clostridia tetani*, *Burkholderia mallei*, *Salmonella* Typhi and *Vibrio vulnificus*	Virus/bacteria	[70,71,72,73,74,75,76]
Mesoporous Silica NPs	Tumor cells	Tumor cells	[77,78]
Virus-like particles	Hepatitis B virus, malaria, *Theileria parva*, *Respiratory Syncytial virus* (RSV), influenza, circoviruses and SARS-CoV-2	virus/parasite	[79,80,81,82,83,84]
Protein-based NPs	SARS-CoV-2	Virus	[85,86]
Self-adjuvanting polyguanidine nanovaccines	Tumor cells	Tumor cells	[87]
Liposomes	Influenza virus, hepatitis A, Human immunosuppressive Virus (HIV), *Pseudomonas aeruginosa*, and methicillin-resistant *Staphylococcus aureus*	Virus/bacteria	[76,88,89,90,91,92]
Silicon scalable lipid NPs	SARS-CoV-2	Virus	[93]
Dendrimer-RNA NPs	H1N1 influenza, Ebola, and *Toxoplasma gondii*	Virus /parasite	[94]
Multiplexed dendrimer with lipid-based NPs	Tumor cells	Tumor cells	[95]
Fluoropolymer-based nanovaccines	Tumor cells	Tumor cells	[96]
Extracellular vesicle-coated multi-antigenic nanovaccines	Multidrug resistant *S. aureus*	Bacteria	[97]
OMV-based nanovaccines	*Escherichia coli* serotype O_2_	Bacteria	[98]
Carbon nanotubes and poly-anhydrous NPs	*Anaplasma marginale* and *Mycobacterium paratuberculosis*	Protozoa/bacteria	[99]
Chitosan	*Salmonella* Enteritidis, *Streptococcus*, avian pathogenic *E*. *coli*, NDV, tuberculosis, *Flavobacterium columnare*, *S. aureus*, *E. coli K1* and *Toxoplasma*	Bacteria/virus/parasite	[100,101,102,103,104,105,106,107,108,109,110]
Polyanhydride NPs	*Bacillus anthracis* and *Yersinia pestis*	Bacteria	[111]
Nanoplasmids	*Trypanosoma cruzi*	Parasite	[112]
Antigen-absorbing silica vesicles	Tick-borne diseases	Tick-borne diseases	[113]
Zinc oxide NPs	*E. coli*, *Salmonella* spp. and H1N1	Bacteria/Virus	[114,115]
Selenium NPs	*S. aureus* and *E. coli*	Bacteria	[116]
Poly (lactic acid-coglycolic acid) polymeric NPs	*C. trachomatis*, *C. pneumoniae*, *Helicobacter pylori*, *Listeria monocytogenes*, *Salmonella* Typhimurium and *Brucella melitensis*	Bacteria	[117,118,119,120,121]
Chitosan and glutamic acid polymeric NPs	*H. pylori*	Bacteria	[122]
Silver NPs (AgNPs)	H3N2, feline calicivirus, infectious bursal disease virus (IBDV), respiratory syncytial virus (RSV), Rift Valley Fever virus (RVF), HIV, adenovirus, poliovirus, norovirus, *Acinetobacter baumannii*, feline coronavirus, *E. coli*, *S. aureus* and *P. aeruginosa*	Virus/bacteria	[123,124,125,126,127,128,129,130,131,132,133,134,135,136]
Dendrimers	*Schistosoma*	Parasite	[137]
Self-assembling polypeptide NPs	Toxoplasmosis	Parasite	[138]
Porous silicon NPs	*P. aeruginosa*	Bacteria	[139]
Calcium-alginate NPs	Methicillin-resistant *S. aureus*	Bacteria	[140]
Ferritin NPs	*Neisseria gonorrhoeae*	Bacteria	[141]
Nanosuspensions	*Cryptosporidium parvum*	Parasite	[142]
Selenium nanoparticle functionalized with oseltamivir	Enterovirus	Virus	[143]
LNP- siVP35-3	EBOV	Virus	[144]
Multivalent peptide–polymer NPs	Influenza	Virus	[145]
Nano-vesicles	*A. marginale* and *shiga toxin by E. coli*	Parasite/bacteria	[146,147]
TMC NPs	Influenza	Virus	[148]

**Table 3:** Examples of lipid-based NP vaccines widely used in preclinical and clinical studies.

Trade Name	Composition	Indication	Ref.
Vaxfectin^®^	Liposomal vaccine	Herpes simplex virus type 2 and influenza virus	[91]
Epaxal^®^	Liposomal vaccine	Hepatitis A infection	[92,159]
Doxil^®^ and Abraxane^®^	Liposomal doxorubicin and albumin-bound paclitaxel	Approved by the FDA and are used in treating Various cancers	[160]
Shingrix^®^	Recombinant VZV glycoprotein E on liposomes carrier	Approved by FDA against herpes virus	[161]
Onivyde^®^	Liposomal irinotecan	Pancreatic cancer	[162]
W_ova1	Liposome-formulated mRNAs	Ovarian cancer	[163]
DPX-0907	Liposomes containing a polynucleotide adjuvant	Ovarian, breast, and prostate cancer	[164]
Lipovaxin-MM	Tumor antigen-containing multicomponent liposomes with the DC-targeting molecule DMS-5000	Metastatic melanoma	[165]
Vyxeos^®^	Liposomal cytarabine and daunorubicin	Acute myeloid leukemia	[166,167]
VaxiSome™	Liposomal adjuvant	Influenza	[168]
MPER-656	HIV-1 gp41 membrane-proximal external region (MPER) on liposome carrier	HIV	[169]
LNP CL-0059/CL-0137	mRNA LNP	RSV	[170]
BNT165b1	mRNA LNP	Malaria	[171]
MRT5413	LNP-formulated, modified mRNA	Influenza	[172]
ARCT-021, MRT5500, Pfizer-BioNTech and Moderna COVID-19, ChulaCov19, mRNA-1273, CVnCoV, LNP-nCoV-saRNA	mRNA LNP	COVID-19	[68,173,174,175,176,177,178,179,180]

## Data Availability

All data were included in the published paper.

## References

[1] Hedman H.D., Krawczyk E., Helmy Y.A., Zhang L., Varga C. (2021). Host Diversity and Potential Transmission Pathways of SARS-CoV-2 at the Human-Animal Interface. Pathogens.

[2] Hemelaar J., Elangovan R., Yun J., Dickson-Tetteh L., Fleminger I., Kirtley S., Williams B., Gouws-Williams E., Ghys P.D., Alash’le G.A. (2019). Global and regional molecular epidemiology of HIV-1, 1990–2015: A systematic review, global survey, and trend analysis. Lancet Infect. Dis..

[3] Ksiazek T.G., Erdman D., Goldsmith C.S., Zaki S.R., Peret T., Emery S., Tong S., Urbani C., Comer J.A., Lim W. (2003). A novel coronavirus associated with severe acute respiratory syndrome. N. Engl. J. Med..

[4] Mehendale R., Joshi M., Patravale V.B. (2013). Nanomedicines for treatment of viral diseases. Crit. Rev. ™ Ther. Drug Carr. Syst..

[5] Salem M., El-Metwally M., Saber W., Negm S., El-Kott A., Mazroua Y., Makhlouf A., Moustafa M. (2022). Secondary antiviral metabolites from fungi with special reference to coronaviruses. Biocell.

[6] Helmy Y.A., Taha-Abdelaziz K., Hawwas H.A.E.-H., Ghosh S., AlKafaas S.S., Moawad M.M.M., Saied E.M., Kassem I.I., Mawad A.M.M. (2023). Antimicrobial Resistance and Recent Alternatives to Antibiotics for the Control of Bacterial Pathogens with an Emphasis on Foodborne Pathogens. Antibiotics.

[7] Kabir A., Lamichhane B., Habib T., Adams A., El-Sheikh Ali H., Slovis N.M., Troedsson M.H.T., Helmy Y.A. (2024). Antimicrobial Resistance in Equines: A Growing Threat to Horse Health and Beyond—A Comprehensive Review. Antibiotics.

[8] Roope L.S., Smith R.D., Pouwels K.B., Buchanan J., Abel L., Eibich P., Butler C.C., Tan P.S., Walker A.S., Robotham J.V. (2019). The challenge of antimicrobial resistance: What economics can contribute. Science.

[9] Lamichhane B., Mawad A.M.M., Saleh M., Kelley W.G., Harrington P.J., Lovestad C.W., Amezcua J., Sarhan M.M., El Zowalaty M.E., Ramadan H. (2024). Salmonellosis: An Overview of Epidemiology, Pathogenesis, and Innovative Approaches to Mitigate the Antimicrobial Resistant Infections. Antibiotics.

[10] Mullins L.P., Mason E., Winter K., Sadarangani M. (2023). Vaccination is an integral strategy to combat antimicrobial resistance. PLoS Pathog..

[11] Fawzy M., Helmy Y.A. (2019). The One Health Approach is Necessary for the Control of Rift Valley Fever Infections in Egypt: A Comprehensive Review. Viruses.

[12] Helmy Y.A., El-Adawy H., Abdelwhab E.M. (2017). A Comprehensive Review of Common Bacterial, Parasitic and Viral Zoonoses at the Human-Animal Interface in Egypt. Pathogens.

[13] Heng W.T., Yew J.S., Poh C.L. (2022). Nanovaccines against viral infectious diseases. Pharmaceutics.

[14] Helmy Y.A., Kassem I.I., Rajashekara G. (2021). Immuno-modulatory effect of probiotic *E. coli* Nissle 1917 in polarized human colonic cells against *Campylobacter jejuni* infection. Gut Microbes.

[15] Zepp F. (2010). Principles of vaccine design—Lessons from nature. Vaccine.

[16] Kiboneka A.N. (2021). Basic concepts in clinical immunology: A review. World J. Adv. Res. Rev..

[17] Sim S., Wong N.K. (2021). Nanotechnology and its use in imaging and drug delivery. Biomed. Rep..

[18] Abdelaziz K., Helmy Y.A., Yitbarek A., Hodgins D.C., Sharafeldin T.A., Selim M.S.H. (2024). Advances in Poultry Vaccines: Leveraging Biotechnology for Improving Vaccine Development, Stability, and Delivery. Vaccines.

[19] Elaish M., Ngunjiri J.M., Ali A., Xia M., Ibrahim M., Jang H., Hiremath J., Dhakal S., Helmy Y.A., Jiang X. (2017). Supplementation of inactivated influenza vaccine with norovirus P particle-M2e chimeric vaccine enhances protection against heterologous virus challenge in chickens. PLoS ONE.

[20] Murugan B., Sagadevan S. (2024). Nano-Vaccines: Opportunities and Challenges in Biomaterial-Based Vaccine Delivery. Biomater. -Inspired Nanomed. Target. Ther..

[21] Peek L.J., Middaugh C.R., Berkland C. (2008). Nanotechnology in vaccine delivery. Adv. Drug Deliv. Rev..

[22] Azharuddin M., Zhu G.H., Sengupta A., Hinkula J., Slater N.K., Patra H.K. (2022). Nano toolbox in immune modulation and nanovaccines. Trends Biotechnol..

[23] Machhi J., Shahjin F., Das S., Patel M., Abdelmoaty M.M., Cohen J.D., Singh P.A., Baldi A., Bajwa N., Kumar R. (2021). Nanocarrier vaccines for SARS-CoV-2. Adv. Drug Deliv. Rev..

[24] Lu W., Cui C., Wang Y., Sun X., Wang S., Yang M., Yu Y., Wang L. (2021). CpG ODN as an adjuvant arouses the vigor of B cells by relieving the negative regulation of surface TLR9 to enhance the antibody response to vaccine. Appl. Microbiol. Biotechnol..

[25] Lozano D., Larraga V., Vallet-Regí M., Manzano M. (2023). An overview of the use of nanoparticles in vaccine development. Nanomaterials.

[26] Fredriksen B.N., Grip J. (2012). PLGA/PLA micro-and nanoparticle formulations serve as antigen depots and induce elevated humoral responses after immunization of Atlantic salmon (*Salmo salar* L.). Vaccine.

[27] Saleh A., Qamar S., Tekin A., Singh R., Kashyap R. (2021). Vaccine development throughout history. Cureus.

[28] Taylor M.W., Taylor M.W. (2014). Smallpox.

[29] Davidson T. (2017). Vaccines: History, Science, and Issues.

[30] Mamelund S.-E. (2008). Influenza, historical. Medicine.

[31] Lombard M., Pastoret P.-P., Moulin A. (2007). A brief history of vaccines and vaccination. Rev. Sci. Tech. Off. Int. Epizoot..

[32] Meeusen E.N., Walker J., Peters A., Pastoret P.-P., Jungersen G. (2007). Current status of veterinary vaccines. Clin. Microbiol. Rev..

[33] Karin H., Lisa B., Damer P. (2018). Vaccines as alternatives to antibiotics for food producing animals. Part 1: Challenges and needs. Vet. Res..

[34] Palomino-Tapia V. (2023). Autogenous Vaccines in the Poultry Industry: A Field Perspective. Poultry Farming-New Perspectives and Applications.

[35] Nagpal G., Usmani S.S., Raghava G.P. (2018). A web resource for designing subunit vaccine against major pathogenic species of bacteria. Front. Immunol..

[36] Malik H., Khan F.H., Ahsan H. (2014). Human papillomavirus: Current status and issues of vaccination. Arch. Virol..

[37] Dai X., Xiong Y., Li N., Jian C. (2019). Vaccine types. Vaccines—The History and Future.

[38] Liljeqvist S., Ståhl S. (1999). Production of recombinant subunit vaccines: Protein immunogens, live delivery systems and nucleic acid vaccines. J. Biotechnol..

[39] Jones R.G., Liu Y., Rigsby P., Sesardic D. (2008). An improved method for development of toxoid vaccines and antitoxins. J. Immunol. Methods.

[40] Andey T., Soni S., Modi S. (2024). Conventional vaccination methods: Inactivated and live attenuated vaccines. Adv. Vaccin. Technol. Infect. Chronic Dis..

[41] Lee S., Nguyen M.T. (2015). Recent advances of vaccine adjuvants for infectious diseases. Immune Netw..

[42] Minor P.D. (2015). Live attenuated vaccines: Historical successes and current challenges. Virology.

[43] Lopez S.M., Sato A.I., Chatterjee A. (2023). Vaccines: An overview. Viral Parasit. Bact. Fungal Infect..

[44] Taha-Abdelaziz K., Singh M., Sharif S., Sharma S., Kulkarni R.R., Alizadeh M., Yitbarek A., Helmy Y.A. (2023). Intervention Strategies to Control *Campylobacter* at Different Stages of the Food Chain. Microorganisms.

[45] Teulon J.-M., Godon C., Chantalat L., Moriscot C., Cambedouzou J., Odorico M., Ravaux J., Podor R., Gerdil A., Habert A. (2018). On the operational aspects of measuring nanoparticle sizes. Nanomaterials.

[46] Cai T., Liu H., Zhang S., Hu J., Zhang L. (2021). Delivery of nanovaccine towards lymphoid organs: Recent strategies in enhancing cancer immunotherapy. J. Nanobiotechnol..

[47] Elattar K.M., Al-Otibi F.O., El-Hersh M.S., Attia A.A., Eldadamony N.M., Elsayed A., Menaa F., Saber W.I. (2024). Multifaceted chemical and bioactive features of Ag@ TiO_2_ and Ag@ SeO_2_ core/shell nanoparticles biosynthesized using *Beta vulgaris* L. extract. Heliyon.

[48] Chattopadhyay S., Chen J.-Y., Chen H.-W., Hu C.-M.J. (2017). Nanoparticle vaccines adopting virus-like features for enhanced immune potentiation. Nanotheranostics.

[49] Zhang W., Wang L., Liu Y., Chen X., Liu Q., Jia J., Yang T., Qiu S., Ma G. (2014). Immune responses to vaccines involving a combined antigen–nanoparticle mixture and nanoparticle-encapsulated antigen formulation. Biomaterials.

[50] Bhardwaj P., Bhatia E., Sharma S., Ahamad N., Banerjee R. (2020). Advancements in prophylactic and therapeutic nanovaccines. Acta Biomater..

[51] Yin Q., Wang Y., Xiang Y., Xu F. (2022). Nanovaccines: Merits, and diverse roles in boosting antitumor immune responses. Hum. Vaccines Immunother..

[52] Li M., Kaminskas L.M., Marasini N. (2021). Recent advances in nano/microparticle-based oral vaccines. J. Pharm. Investig..

[53] Sabourian P., Yazdani G., Ashraf S.S., Frounchi M., Mashayekhan S., Kiani S., Kakkar A. (2020). Effect of physico-chemical properties of nanoparticles on their intracellular uptake. Int. J. Mol. Sci..

[54] González-García L.E., MacGregor M.N., Visalakshan R.M., Lazarian A., Cavallaro A.A., Morsbach S., Mierczynska-Vasilev A., Mailänder V., Landfester K., Vasilev K. (2022). Nanoparticles surface chemistry influence on protein corona composition and inflammatory responses. Nanomaterials.

[55] Diaz-Arévalo D., Zeng M. (2020). Nanoparticle-based vaccines: Opportunities and limitations. Nanopharmaceuticals.

[56] Kirtane A.R., Verma M., Karandikar P., Furin J., Langer R., Traverso G. (2021). Nanotechnology approaches for global infectious diseases. Nat. Nanotechnol..

[57] Gregory A.E., Titball R., Williamson D. (2013). Vaccine delivery using nanoparticles. Front. Cell. Infect. Microbiol..

[58] Manju K., Raj S.N., Ranjini H., Nayaka S.C., Ashwini P., Satish S., Prasad M.N., Chouhan R.S., Baker S. (2023). Nanovaccines to combat drug resistance: The next-generation immunisation. Future J. Pharm. Sci..

[59] Kheirollahpour M., Mehrabi M., Dounighi N.M., Mohammadi M., Masoudi A. (2020). Nanoparticles and vaccine development. Pharm. Nanotechnol..

[60] Liu J., Zhang R., Xu Z.P. (2019). Nanoparticle-based nanomedicines to promote cancer immunotherapy: Recent advances and future directions. Small.

[61] Kumru O.S., Joshi S.B., Smith D.E., Middaugh C.R., Prusik T., Volkin D.B. (2014). Vaccine instability in the cold chain: Mechanisms, analysis and formulation strategies. Biologicals.

[62] Torres-Sangiao E., Holban A.M., Gestal M.C. (2016). Advanced nanobiomaterials: Vaccines, diagnosis and treatment of infectious diseases. Molecules.

[63] Abusalah M.A.H., Chopra H., Sharma A., Mustafa S.A., Choudhary O.P., Sharma M., Dhawan M., Khosla R., Loshali A., Sundriyal A. (2023). Nanovaccines: A game changing approach in the fight against infectious diseases. Biomed. Pharmacother..

[64] Jazayeri S.D., Lim H.X., Shameli K., Yeap S.K., Poh C.L. (2021). Nano and microparticles as potential oral vaccine carriers and adjuvants against infectious diseases. Front. Pharmacol..

[65] Rosenbaum P., Tchitchek N., Joly C., Rodriguez Pozo A., Stimmer L., Langlois S., Hocini H., Gosse L., Pejoski D., Cosma A. (2021). Vaccine inoculation route modulates early immunity and consequently antigen-specific immune response. Front. Immunol..

[66] Xie S., Pan B., Wang M., Zhu L., Wang F., Dong Z., Wang X., Zhou W. (2010). Formulation, characterization and pharmacokinetics of praziquantel-loaded hydrogenated castor oil solid lipid nanoparticles. Nanomedicine.

[67] Heidari-Kharaji M., Taheri T., Doroud D., Habibzadeh S., Badirzadeh A., Rafati S. (2016). Enhanced paromomycin efficacy by solid lipid nanoparticle formulation against Leishmania in mice model. Parasite Immunol..

[68] Thi T.T.H., Suys E.J., Lee J.S., Nguyen D.H., Park K.D., Truong N.P. (2021). Lipid-based nanoparticles in the clinic and clinical trials: From cancer nanomedicine to COVID-19 vaccines. Vaccines.

[69] Kondel R., Shafiq N., Kaur I.P., Singh M.P., Pandey A.K., Ratho R.K., Malhotra S. (2019). Effect of acyclovir solid lipid nanoparticles for the treatment of herpes simplex virus (HSV) infection in an animal model of HSV-1 infection. Pharm. Nanotechnol..

[70] Lee B., Park J., Ryu M., Kim S., Joo M., Yeom J.-H., Kim S., Park Y., Lee K., Bae J. (2017). Antimicrobial peptide-loaded gold nanoparticle-DNA aptamer conjugates as highly effective antibacterial therapeutics against *Vibrio vulnificus*. Sci. Rep..

[71] Chowdhury R., Ilyas H., Ghosh A., Ali H., Ghorai A., Midya A., Jana N.R., Das S., Bhunia A. (2017). Multivalent gold nanoparticle–peptide conjugates for targeting intracellular bacterial infections. Nanoscale.

[72] Gregory A.E., Judy B.M., Qazi O., Blumentritt C.A., Brown K.A., Shaw A.M., Torres A.G., Titball R.W. (2015). A gold nanoparticle-linked glycoconjugate vaccine against *Burkholderia mallei*. Nanomed. Nanotechnol. Biol. Med..

[73] Chen Y.-S., Hung Y.-C., Lin W.-H., Huang G.S. (2010). Assessment of gold nanoparticles as a size-dependent vaccine carrier for enhancing the antibody response against synthetic foot-and-mouth disease virus peptide. Nanotechnology.

[74] Lee M.-Y., Yang J.-A., Jung H.S., Beack S., Choi J.E., Hur W., Koo H., Kim K., Yoon S.K., Hahn S.K. (2012). Hyaluronic acid–gold nanoparticle/interferon α complex for targeted treatment of hepatitis C virus infection. ACS Nano.

[75] Farfán-Castro S., García-Soto M.J., Betancourt-Mendiola L., Cervantes J., Segura R., González-Ortega O., Rosales-Mendoza S. (2024). Synthesis and evaluation of gold nanoparticles conjugated with five antigenic peptides derived from the spike protein of SARS-CoV-2 for vaccine development. Front. Nanotechnol..

[76] Halwani M., Yebio B., Suntres Z., Alipour M., Azghani A., Omri A. (2008). Co-encapsulation of gallium with gentamicin in liposomes enhances antimicrobial activity of gentamicin against *Pseudomonas aeruginosa*. J. Antimicrob. Chemother..

[77] Theivendran S., Lazarev S., Yu C. (2023). Mesoporous silica/organosilica nanoparticles for cancer immunotherapy. Exploration.

[78] Song C., Li F., Wang S., Wang J., Wei W., Ma G. (2020). Recent advances in particulate adjuvants for cancer vaccination. Adv. Ther..

[79] Madapong A., Petro-Turnquist E.M., Webby R.J., McCormick A.A., Weaver E.A. (2024). Immunity and Protective Efficacy of a Plant-Based Tobacco Mosaic Virus-like Nanoparticle Vaccine against Influenza a Virus in Mice. Vaccines.

[80] Huertas-Díaz M.C., Phan S., Elson A., Nuñez I., Wei H., Sakamoto K., He B. (2019). Parainfluenza virus 5 (PIV5) amplifying virus-like particles expressing respiratory syncytial virus (RSV) antigens protect mice against RSV infection. Vaccine.

[81] Lacasta A., Mody K.T., De Goeyse I., Yu C., Zhang J., Nyagwange J., Mwalimu S., Awino E., Saya R., Njoroge T. (2021). Synergistic effect of two nanotechnologies enhances the protective capacity of the *Theileria parva* sporozoite p67C antigen in cattle. J. Immunol..

[82] Tariq H., Batool S., Asif S., Ali M., Abbasi B.H. (2022). Virus-like particles: Revolutionary platforms for developing vaccines against emerging infectious diseases. Front. Microbiol..

[83] Dai S., Wang H., Deng F. (2018). Advances and challenges in enveloped virus-like particle (VLP)-based vaccines. J. Immunol. Sci..

[84] Michel M.-L., Tiollais P. (2010). Hepatitis B vaccines: Protective efficacy and therapeutic potential. Pathol. Biol..

[85] Keech C., Albert G., Cho I., Robertson A., Reed P., Neal S., Plested J.S., Zhu M., Cloney-Clark S., Zhou H. (2020). Phase 1–2 trial of a SARS-CoV-2 recombinant spike protein nanoparticle vaccine. N. Engl. J. Med..

[86] Vu M.N., Kelly H.G., Kent S.J., Wheatley A.K. (2021). Current and future nanoparticle vaccines for COVID-19. EBioMedicine.

[87] Liao Z., Huang J., Lo P.-C., Lovell J.F., Jin H., Yang K. (2022). Self-adjuvanting cancer nanovaccines. J. Nanobiotechnol..

[88] Li Y., Su T., Zhang Y., Huang X., Li J., Li C. (2015). Liposomal co-delivery of daptomycin and clarithromycin at an optimized ratio for treatment of methicillin-resistant *Staphylococcus aureus* infection. Drug Deliv..

[89] Wang J., Li P., Yu Y., Fu Y., Jiang H., Lu M., Sun Z., Jiang S., Lu L., Wu M.X. (2020). Pulmonary surfactant–biomimetic nanoparticles potentiate heterosubtypic influenza immunity. Science.

[90] Hanson M.C., Abraham W., Crespo M.P., Chen S.H., Liu H., Szeto G.L., Kim M., Reinherz E.L., Irvine D.J. (2015). Liposomal vaccines incorporating molecular adjuvants and intrastructural T-cell help promote the immunogenicity of HIV membrane-proximal external region peptides. Vaccine.

[91] Smith L.R., Wloch M.K., Ye M., Reyes L.R., Boutsaboualoy S., Dunne C.E., Chaplin J.A., Rusalov D., Rolland A.P., Fisher C.L. (2010). Phase 1 clinical trials of the safety and immunogenicity of adjuvanted plasmid DNA vaccines encoding influenza A virus H5 hemagglutinin. Vaccine.

[92] Bovier P.A. (2008). Epaxal^®^: A virosomal vaccine to prevent hepatitis A infection. Expert Rev. Vaccines.

[93] Huang X., Ma Y., Ma G., Xia Y. (2024). Unlocking the therapeutic applicability of LNP-mRNA: Chemistry, formulation, and clinical strategies. Research.

[94] Chahal J.S., Khan O.F., Cooper C.L., McPartlan J.S., Tsosie J.K., Tilley L.D., Sidik S.M., Lourido S., Langer R., Bavari S. (2016). Dendrimer-RNA nanoparticles generate protective immunity against lethal Ebola, H1N1 influenza, and *Toxoplasma gondii* challenges with a single dose. Proc. Natl. Acad. Sci. USA.

[95] Zhang D., Wang G., Yu X., Wei T., Farbiak L., Johnson L.T., Taylor A.M., Xu J., Hong Y., Zhu H. (2022). Enhancing CRISPR/Cas gene editing through modulating cellular mechanical properties for cancer therapy. Nat. Nanotechnol..

[96] Xu J., Lv J., Zhuang Q., Yang Z., Cao Z., Xu L., Pei P., Wang C., Wu H., Dong Z. (2020). A general strategy towards personalized nanovaccines based on fluoropolymers for post-surgical cancer immunotherapy. Nat. Nanotechnol..

[97] Chen G., Bai Y., Li Z., Wang F., Fan X., Zhou X. (2020). Bacterial extracellular vesicle-coated multi-antigenic nanovaccines protect against drug-resistant *Staphylococcus aureus* infection by modulating antigen processing and presentation pathways. Theranostics.

[98] Hu R., Liu H., Wang M., Li J., Lin H., Liang M., Gao Y., Yang M. (2020). An OMV-based nanovaccine confers safety and protection against pathogenic *Escherichia coli* via both humoral and predominantly Th1 immune responses in poultry. Nanomaterials.

[99] Thukral A., Ross K., Hansen C., Phanse Y., Narasimhan B., Steinberg H., Talaat A.M. (2020). A single dose polyanhydride-based nanovaccine against paratuberculosis infection. npj Vaccines.

[100] Etewa S.E., El-Maaty D.A.A., Hamza R.S., Metwaly A.S., Sarhan M.H., Abdel-Rahman S.A., Fathy G.M., El-Shafey M.A. (2018). Assessment of spiramycin-loaded chitosan nanoparticles treatment on acute and chronic toxoplasmosis in mice. J. Parasit. Dis..

[101] Zhang J., Sun H., Gao C., Wang Y., Cheng X., Yang Y., Gou Q., Lei L., Chen Y., Wang X. (2021). Development of a chitosan--modified PLGA nanoparticle vaccine for protection against *Escherichia coli* K1 caused meningitis in mice. J. Nanobiotechnol..

[102] Tao J., Zhang Y., Shen A., Yang Y., Diao L., Wang L., Cai D., Hu Y. (2020). Injectable chitosan-based thermosensitive hydrogel/nanoparticle-loaded system for local delivery of vancomycin in the treatment of osteomyelitis. Int. J. Nanomed..

[103] Kitiyodom S., Trullàs C., Rodkhum C., Thompson K.D., Katagiri T., Temisak S., Namdee K., Yata T., Pirarat N. (2021). Modulation of the mucosal immune response of red tilapia (*Oreochromis* sp.) against columnaris disease using a biomimetic-mucoadhesive nanovaccine. Fish Shellfish. Immunol..

[104] El-Sissi A.F., Mohamed F.H., Danial N.M., Gaballah A.Q., Ali K.A. (2020). Chitosan and chitosan nanoparticles as adjuvant in local Rift Valley Fever inactivated vaccine. 3 Biotech.

[105] Nevagi R.J., Khalil Z.G., Hussein W.M., Powell J., Batzloff M.R., Capon R.J., Good M.F., Skwarczynski M., Toth I. (2018). Polyglutamic acid-trimethyl chitosan-based intranasal peptide nano-vaccine induces potent immune responses against group A streptococcus. Acta Biomater..

[106] Zhao K., Chen G., Shi X.-m., Gao T.-t., Li W., Zhao Y., Zhang F.-q., Wu J., Cui X., Wang Y.-F. (2012). Preparation and efficacy of a live newcastle disease virus vaccine encapsulated in chitosan nanoparticles. PLoS ONE.

[107] Das I., Padhi A., Mukherjee S., Dash D.P., Kar S., Sonawane A. (2017). Biocompatible chitosan nanoparticles as an efficient delivery vehicle for *Mycobacterium tuberculosis* lipids to induce potent cytokines and antibody response through activation of γδ T cells in mice. Nanotechnology.

[108] Mohammed G.M., ElZorkany H.E., Farroh K.Y., Abd El-Aziz W.R., Elshoky H.A. (2021). Potential improvement of the immune response of chickens against *E. coli* vaccine by using two forms of chitosan nanoparticles. Int. J. Biol. Macromol..

[109] Renu S., Renukaradhya G.J. (2020). Chitosan nanoparticle based mucosal vaccines delivered against infectious diseases of poultry and pigs. Front. Bioeng. Biotechnol..

[110] Acevedo-Villanueva K., Renu S., Gourapura R., Selvaraj R. (2021). Efficacy of a nanoparticle vaccine administered in-ovo against *Salmonella* in broilers. PLoS ONE.

[111] Kelly S.M., Larsen K.R., Darling R., Petersen A.C., Bellaire B.H., Wannemuehler M.J., Narasimhan B. (2021). Single-dose combination nanovaccine induces both rapid and durable humoral immunity and toxin neutralizing antibody responses against *Bacillus anthracis*. Vaccine.

[112] Chowdhury I.H., Lokugamage N., Garg N.J. (2020). Experimental nanovaccine offers protection against repeat exposures *to Trypanosoma cruzi* through activation of polyfunctional T cell response. Front. Immunol..

[113] Mody K.T., Zhang B., Li X., Fletcher N.L., Akhter D.T., Jarrett S., Zhang J., Yu C., Thurecht K.J., Mahony T.J. (2021). Characterization of the biodistribution of a silica vesicle nanovaccine carrying a *Rhipicephalus* (*Boophilus*) microplus protective antigen with in vivo live animal imaging. Front. Bioeng. Biotechnol..

[114] Ghaffari H., Tavakoli A., Moradi A., Tabarraei A., Bokharaei-Salim F., Zahmatkeshan M., Farahmand M., Javanmard D., Kiani S.J., Esghaei M. (2019). Inhibition of H1N1 influenza virus infection by zinc oxide nanoparticles: Another emerging application of nanomedicine. J. Biomed. Sci..

[115] Rajeshkumar S., Bharath L. (2018). Controlling of food borne pathogens by nanoparticles. Bioorganic Phase Nat. Food Overv..

[116] Vinayamohan P.G., Joseph D., Viju L.S., Venkitanarayanan K. (2023). Efficacy of selenium for controlling infectious diseases. Selenium and Human Health.

[117] Maleki M., Salouti M., Shafiee Ardestani M., Talebzadeh A. (2019). Preparation of a nanovaccine against Brucella melitensis M16 based on PLGA nanoparticles and oligopolysaccharide antigen. Artif. Cells Nanomed. Biotechnol..

[118] Lee J.-A., Jung B.-G., Kim T.-H., Kim Y.-M., Park M.-H., Hyun P.-m., Jeon J.-w., Park J.-k., Cho C.-W., Suh G.-H. (2014). Poly d, l-lactide-co-glycolide (PLGA) nanoparticle-encapsulated honeybee (*Apis melifera*) venom promotes clearance of *Salmonella enterica serovar Typhimurium* infection in experimentally challenged pigs through the up-regulation of T helper type 1 specific immune responses. Vet. Immunol. Immunopathol..

[119] Demento S.L., Cui W., Criscione J.M., Stern E., Tulipan J., Kaech S.M., Fahmy T.M. (2012). Role of sustained antigen release from nanoparticle vaccines in shaping the T cell memory phenotype. Biomaterials.

[120] Tan Z., Liu W., Liu H., Li C., Zhang Y., Meng X., Tang T., Xi T., Xing Y. (2017). Oral Helicobacter pylori vaccine-encapsulated acid-resistant HP55/PLGA nanoparticles promote immune protection. Eur. J. Pharm. Biopharm..

[121] Toti U.S., Guru B.R., Hali M., McPharlin C.M., Wykes S.M., Panyam J., Whittum-Hudson J.A. (2011). Targeted delivery of antibiotics to intracellular chlamydial infections using PLGA nanoparticles. Biomaterials.

[122] Ramteke S., Ganesh N., Bhattacharya S., Jain N.K. (2009). Amoxicillin, clarithromycin, and omeprazole based targeted nanoparticles for the treatment of *H. pylori*. J. Drug Target..

[123] Ardestani M.S., Fordoei A.S., Abdoli A., Ahangari Cohan R., Bahramali G., Sadat S.M., Siadat S.D., Moloudian H., Nassiri Koopaei N., Bolhasani A. (2015). Nanosilver based anionic linear globular dendrimer with a special significant antiretroviral activity. J. Mater. Sci. Mater. Med..

[124] Park S., Park H.H., Kim S.Y., Kim S.J., Woo K., Ko G. (2014). Antiviral properties of silver nanoparticles on a magnetic hybrid colloid. Appl. Environ. Microbiol..

[125] Huy T.Q., Thanh N.T.H., Thuy N.T., Van Chung P., Hung P.N., Le A.-T., Hanh N.T.H. (2017). Cytotoxicity and antiviral activity of electrochemical–synthesized silver nanoparticles against poliovirus. J. Virol. Methods.

[126] Chen N., Zheng Y., Yin J., Li X., Zheng C. (2013). Inhibitory effects of silver nanoparticles against adenovirus type 3 in vitro. J. Virol. Methods.

[127] Sanchez-Villamil J.I., Tapia D., Torres A.G. (2019). Development of a gold nanoparticle vaccine against enterohemorrhagic *Escherichia coli* O157: H7. mBio.

[128] Chen Y.-N., Hsueh Y.-H., Hsieh C.-T., Tzou D.-Y., Chang P.-L. (2016). Antiviral activity of graphene–silver nanocomposites against non-enveloped and enveloped viruses. Int. J. Environ. Res. Public Health.

[129] Chen P., Wu Z., Leung A., Chen X., Landao-Bassonga E., Gao J., Chen L., Zheng M., Yao F., Yang H. (2018). Fabrication of a silver nanoparticle-coated collagen membrane with anti-bacterial and anti-inflammatory activities for guided bone regeneration. Biomed. Mater..

[130] Baram-Pinto D., Shukla S., Perkas N., Gedanken A., Sarid R. (2009). Inhibition of herpes simplex virus type 1 infection by silver nanoparticles capped with mercaptoethane sulfonate. Bioconjugate Chem..

[131] Tiwari V., Tiwari M., Solanki V. (2017). Polyvinylpyrrolidone-capped silver nanoparticle inhibits infection of carbapenem-resistant strain of *Acinetobacter baumannii* in the human pulmonary epithelial cell. Front. Immunol..

[132] Borrego B., Lorenzo G., Mota-Morales J.D., Almanza-Reyes H., Mateos F., López-Gil E., de la Losa N., Burmistrov V.A., Pestryakov A.N., Brun A. (2016). Potential application of silver nanoparticles to control the infectivity of Rift Valley fever virus in vitro and in vivo. Nanomed. Nanotechnol. Biol. Med..

[133] Yang X.X., Li C.M., Huang C.Z. (2016). Curcumin modified silver nanoparticles for highly efficient inhibition of respiratory syncytial virus infection. Nanoscale.

[134] Pangestika R., Ernawati R. (2017). Antiviral activity effect of silver nanoparticles (Agnps) solution against the growth of infectious bursal disease virus on embryonated chicken eggs with Elisa test. KnE Life Sci..

[135] Kuppan G., Sangeeta K. (2016). Dose and Size-Dependent Antiviral Effects of Silver Nanoparticles on Feline Calicivirus, a Human Norovirus Surrogate. Foodborne Pathog Dis..

[136] Xiang D., Zheng Y., Duan W., Li X., Yin J., Shigdar S., O’Connor M.L., Marappan M., Zhao X., Miao Y. (2013). Inhibition of A/Human/Hubei/3/2005 (H3N2) influenza virus infection by silver nanoparticles in vitro and in vivo. Int. J. Nanomed..

[137] Folliero V., Zannella C., Chianese A., Stelitano D., Ambrosino A., De Filippis A., Galdiero M., Franci G., Galdiero M. (2021). Application of dendrimers for treating parasitic diseases. Pharmaceutics.

[138] El Bissati K., Zhou Y., Paulillo S.M., Raman S.K., Karch C.P., Roberts C.W., Lanar D.E., Reed S., Fox C., Carter D. (2017). Protein nanovaccine confers robust immunity against Toxoplasma. npj Vaccines.

[139] Kwon E.J., Skalak M., Bertucci A., Braun G., Ricci F., Ruoslahti E., Sailor M.J., Bhatia S.N. (2017). Porous silicon nanoparticle delivery of tandem peptide anti-infectives for the treatment of Pseudomonas aeruginosa lung infections. Adv. Mater..

[140] Gowri M., Latha N., Suganya K., Murugan M., Rajan M. (2021). Calcium alginate nanoparticle crosslinked phosphorylated polyallylamine to the controlled release of clindamycin for osteomyelitis treatment. Drug Dev. Ind. Pharm..

[141] Wang L., Xing D., Le Van A., Jerse A.E., Wang S. (2017). Structure-based design of ferritin nanoparticle immunogens displaying antigenic loops of *Neisseria gonorrhoeae*. FEBS Open Bio.

[142] Lemke A., Kiderlen A.F., Petri B., Kayser O. (2010). Delivery of amphotericin B nanosuspensions to the brain and determination of activity against *Balamuthia mandrillaris amebas*. Nanomed. Nanotechnol. Biol. Med..

[143] Zhong J., Xia Y., Hua L., Liu X., Xiao M., Xu T., Zhu B., Cao H. (2019). Functionalized selenium nanoparticles enhance the anti-EV71 activity of oseltamivir in human astrocytoma cell model. Artif. Cells Nanomed. Biotechnol..

[144] Thi E.P., Mire C.E., Lee A.C., Geisbert J.B., Zhou J.Z., Agans K.N., Snead N.M., Deer D.J., Barnard T.R., Fenton K.A. (2015). Lipid nanoparticle siRNA treatment of Ebola-virus-Makona-infected nonhuman primates. Nature.

[145] Lauster D., Glanz M., Bardua M., Ludwig K., Hellmund M., Hoffmann U., Hamann A., Böttcher C., Haag R., Hackenberger C.P. (2017). Multivalent peptide–nanoparticle conjugates for influenza-virus inhibition. Angew. Chem. Int. Ed..

[146] Rodrigues-Jesus M., Fotoran W.L., Cardoso R.M., Araki K., Wunderlich G., Ferreira L.C. (2019). Nano-multilamellar lipid vesicles (NMVs) enhance protective antibody responses against Shiga toxin (Stx2a) produced by enterohemorrhagic *Escherichia coli* strains (EHEC). Braz. J. Microbiol..

[147] Zhang B., Cavallaro A.S., Mody K.T., Zhang J., Deringer J.R., Brown W.C., Mahony T.J., Yu C., Mitter N. (2016). Nanoparticle-based delivery of *Anaplasma marginale* membrane proteins; virb9-1 and virb10 produced in the *Pichia pastoris* expression system. Nanomaterials.

[148] Rungrojcharoenkit K., Sunintaboon P., Ellison D., Macareo L., Midoeng P., Chaisuwirat P., Fernandez S., Ubol S. (2020). Development of an adjuvanted nanoparticle vaccine against influenza virus, an in vitro study. PLoS ONE.

[149] Riehemann K., Schneider S.W., Luger T.A., Godin B., Ferrari M., Fuchs H. (2009). Nanomedicine—Challenge and perspectives. Angew. Chem. Int. Ed..

[150] Lung P., Yang J., Li Q. (2020). Nanoparticle formulated vaccines: Opportunities and challenges. Nanoscale.

[151] Verma M., Ozer I., Xie W., Gallagher R., Teixeira A., Choy M. (2023). The landscape for lipid-nanoparticle-based genomic medicines. Nat. Rev. Drug Discov..

[152] Uddin M.N., Roni M.A. (2021). Challenges of storage and stability of mRNA-based COVID-19 vaccines. Vaccines.

[153] Pal K., Chakroborty S., Nath N. (2022). Limitations of nanomaterials insights in green chemistry sustainable route: Review on novel applications. Green Process. Synth..

[154] Chen S., Huang X., Xue Y., Álvarez-Benedicto E., Shi Y., Chen W., Koo S., Siegwart D.J., Dong Y., Tao W. (2023). Nanotechnology-based mRNA vaccines. Nat. Rev. Methods Primers.

[155] Look M., Bandyopadhyay A., Blum J.S., Fahmy T.M. (2010). Application of nanotechnologies for improved immune response against infectious diseases in the developing world. Adv. Drug Deliv. Rev..

[156] Khan I., Saeed K., Khan I. (2019). Nanoparticles: Properties, applications and toxicities. Arab. J. Chem..

[157] Torchilin V.P. (2005). Recent advances with liposomes as pharmaceutical carriers. Nat. Rev. Drug Discov..

[158] Helmy Y.A., Fawzy M., Elaswad A., Sobieh A., Kenney S.P., Shehata A.A. (2020). The COVID-19 Pandemic: A Comprehensive Review of Taxonomy, Genetics, Epidemiology, Diagnosis, Treatment, and Control. J. Clin. Med..

[159] Heath P.T., Galiza E.P., Baxter D.N., Boffito M., Browne D., Burns F., Chadwick D.R., Clark R., Cosgrove C., Galloway J. (2021). Safety and efficacy of NVX-CoV2373 COVID-19 vaccine. N. Engl. J. Med..

[160] Wang F., Porter M., Konstantopoulos A., Zhang P., Cui H. (2017). Preclinical development of drug delivery systems for paclitaxel-based cancer chemotherapy. J. Control. Release.

[161] Smith J.F., Brownlow M., Brown M., Kowalski R., Esser M.T., Ruiz W., Barr E., Brown D.R., Bryan J.T. (2007). Antibodies from women immunized with Gardasil^®^ cross-neutralize HPV 45 pseudovirions. Hum. Vaccines.

[162] Passero Jr F.C., Grapsa D., Syrigos K.N., Saif M.W. (2016). The safety and efficacy of Onivyde (irinotecan liposome injection) for the treatment of metastatic pancreatic cancer following gemcitabine-based therapy. Expert Rev. Anticancer. Ther..

[163] Kantoff P.W., Higano C.S., Shore N.D., Berger E.R., Small E.J., Penson D.F., Redfern C.H., Ferrari A.C., Dreicer R., Sims R.B. (2010). Sipuleucel-T immunotherapy for castration-resistant prostate cancer. N. Engl. J. Med..

[164] Nordquist L.T., Shore N.D., Elist J.J., Oliver J.C., Gannon W., Shahlaee A.H., Fuller S.A., Ghanbari H.A. (2018). Phase 1 open-label trial to evaluate the safety and immunogenicity of PAN-301-1, a novel nanoparticle therapeutic vaccine, in patients with biochemically relapsed prostate cancer. J. Clin. Oncol..

[165] Lee D.-H., Choi S., Park Y., Jin H.-s. (2021). Mucin1 and Mucin16: Therapeutic targets for cancer therapy. Pharmaceuticals.

[166] Wicki A., Witzigmann D., Balasubramanian V., Huwyler J. (2015). Nanomedicine in cancer therapy: Challenges, opportunities, and clinical applications. J. Control. Release.

[167] Rodríguez F., Caruana P., De la Fuente N., Español P., Gámez M., Balart J., Llurba E., Rovira R., Ruiz R., Martín-Lorente C. (2022). Nano-based approved pharmaceuticals for cancer treatment: Present and future challenges. Biomolecules.

[168] Cech P.G., Aebi T., Abdallah M.S., Mpina M., Machunda E.B., Westerfeld N., Stoffel S.A., Zurbriggen R., Pluschke G., Tanner M. (2011). Virosome-formulated *Plasmodium falciparum* AMA-1 & CSP derived peptides as malaria vaccine: Randomized phase 1b trial in semi-immune adults & children. PLoS ONE.

[169] Porras C., Sampson J.N., Herrero R., Gail M.H., Cortés B., Hildesheim A., Cyr J., Romero B., Schiller J.T., Montero C. (2022). Rationale and design of a double-blind randomized non-inferiority clinical trial to evaluate one or two doses of vaccine against human papillomavirus including an epidemiologic survey to estimate vaccine efficacy: The Costa Rica ESCUDDO trial. Vaccine.

[170] Topalidou X., Kalergis A.M., Papazisis G. (2023). Respiratory syncytial virus vaccines: A review of the candidates and the approved vaccines. Pathogens.

[171] Lamb Y.N. (2021). BNT162b2 mRNA COVID-19 vaccine: First approval. Drugs.

[172] Bhangde S., Lodaya R.N., Amiji M.M. (2023). Nanoscale Vaccines for Influenza. Nanomedicines for the Prevention and Treatment of Infectious Diseases.

[173] Low J.G., De Alwis R., Chen S., Kalimuddin S., Leong Y.S., Mah T.K.L., Yuen N., Tan H.C., Zhang S.L., Sim J.X. (2022). A phase I/II randomized, double-blinded, placebo-controlled trial of a self-amplifying COVID-19 mRNA vaccine. npj Vaccines.

[174] Al L.I.R., Sönmezer M.Ç., Ünal S. (2021). RNA-Based COVID-19 vaccine candidates with clinical phase trials in progress. Turk. J. Med. Sci..

[175] Kalnin K.V., Plitnik T., Kishko M., Zhang J., Zhang D., Beauvais A., Anosova N.G., Tibbitts T., DiNapoli J., Ulinski G. (2021). Immunogenicity and efficacy of mRNA COVID-19 vaccine MRT5500 in preclinical animal models. NPJ Vaccines.

[176] Prompetchara E., Ketloy C., Alameh M.-G., Tharakhet K., Kaewpang P., Yostrerat N., Pitakpolrat P., Buranapraditkun S., Manopwisedjaroen S., Thitithanyanont A. (2023). Immunogenicity and protective efficacy of SARS-CoV-2 mRNA vaccine encoding secreted non-stabilized spike in female mice. Nat. Commun..

[177] World Health Organization (2020). Emergency use designation of COVID-19 candidate vaccines: Ethical considerations for current and future COVID-19 placebo-controlled vaccine trials and trial unblinding: Policy brief, 18 December 2020. Emergency Use Designation of COVID-19 Candidate Vaccines: Ethical Considerations for Current and Future COVID-19 Placebo-Controlled Vaccine Trials and Trial Unblinding: Policy Brief, 18 December 2020.

[178] Shin M.D., Shukla S., Chung Y.H., Beiss V., Chan S.K., Ortega-Rivera O.A., Wirth D.M., Chen A., Sack M., Pokorski J.K. (2020). COVID-19 vaccine development and a potential nanomaterial path forward. Nat. Nanotechnol..

[179] Silva-Pilipich N., Beloki U., Salaberry L., Smerdou C. (2024). Self-Amplifying RNA: A Second Revolution of mRNA Vaccines against COVID-19. Vaccines.

[180] Rauch S., Roth N., Schwendt K., Fotin-Mleczek M., Mueller S.O., Petsch B. (2021). mRNA-based SARS-CoV-2 vaccine candidate CVnCoV induces high levels of virus-neutralising antibodies and mediates protection in rodents. npj Vaccines.

[181] Moon J.J., Suh H., Bershteyn A., Stephan M.T., Liu H., Huang B., Sohail M., Luo S., Ho Um S., Khant H. (2011). Interbilayer-crosslinked multilamellar vesicles as synthetic vaccines for potent humoral and cellular immune responses. Nat. Mater..

[182] Moon J.J., Suh H., Li A.V., Ockenhouse C.F., Yadava A., Irvine D.J. (2012). Enhancing humoral responses to a malaria antigen with nanoparticle vaccines that expand Tfh cells and promote germinal center induction. Proc. Natl. Acad. Sci. USA.

[183] Vickers K.C., Palmisano B.T., Shoucri B.M., Shamburek R.D., Remaley A.T. (2011). MicroRNAs are transported in plasma and delivered to recipient cells by high-density lipoproteins. Nat. Cell Biol..

[184] Moon J.J., Huang B., Irvine D.J. (2012). Engineering nano- and microparticles to tune immunity. Adv. Mater..

[185] Chong P., Huang J.-H., Leng C.-H., Liu S.-J., Chen H.-W. (2015). Recombinant lipoproteins as novel vaccines with intrinsic adjuvant. Adv. Protein Chem. Struct. Biol..

[186] Waheed I., Ali A., Tabassum H., Khatoon N., Lai W.-F., Zhou X. (2024). Lipid-based nanoparticles as drug delivery carriers for cancer therapy. Front. Oncol..

[187] John R., Monpara J., Swaminathan S., Kalhapure R. (2024). Chemistry and art of developing lipid nanoparticles for biologics delivery: Focus on development and scale-up. Pharmaceutics.

[188] Ndeupen S., Qin Z., Jacobsen S., Bouteau A., Estanbouli H., Igyártó B.Z. (2021). The mRNA-LNP platform’s lipid nanoparticle component used in preclinical vaccine studies is highly inflammatory. iScience.

[189] Hou X., Zaks T., Langer R., Dong Y. (2021). Lipid nanoparticles for mRNA delivery. Nat. Rev. Mater..

[190] Xu F., Yuan Y., Wang Y., Yin Q. (2023). Emerging peptide-based nanovaccines: From design synthesis to defense against cancer and infection. Biomed. Pharmacother..

[191] Abdul Ghaffar K., Kumar Giddam A., Zaman M., Skwarczynski M., Toth I. (2014). Liposomes as nanovaccine delivery systems. Curr. Top. Med. Chem..

[192] Tretiakova D., Vodovozova E. (2022). Liposomes as adjuvants and vaccine delivery systems. Biochem. (Mosc.) Suppl. Ser. A Membr. Cell Biol..

[193] Allison A., Gregoriadis G. (1974). Liposomes as immunological adjuvants. Nature.

[194] Yanasarn N., Sloat B.R., Cui Z. (2011). Negatively charged liposomes show potent adjuvant activity when simply admixed with protein antigens. Mol. Pharm..

[195] Hatakeyama H., Akita H., Harashima H. (2013). The polyethyleneglycol dilemma: Advantage and disadvantage of PEGylation of liposomes for systemic genes and nucleic acids delivery to tumors. Biol. Pharm. Bull..

[196] Shetye L., Sherlekar A., Mendhulkar V. (2023). Liposome-Based Drug Delivery—A New Therapeutic Paradigm. Advanced Drug Delivery: Methods and Applications.

[197] Gordillo-Galeano A., Mora-Huertas C.E. (2018). Solid lipid nanoparticles and nanostructured lipid carriers: A review emphasizing on particle structure and drug release. Eur. J. Pharm. Biopharm..

[198] Pensado A., Seijo B., Sanchez A. (2014). Current strategies for DNA therapy based on lipid nanocarriers. Expert Opin. Drug Deliv..

[199] Peres L.B., Peres L.B., de Araújo P.H.H., Sayer C. (2016). Solid lipid nanoparticles for encapsulation of hydrophilic drugs by an organic solvent free double emulsion technique. Colloids Surf. B Biointerfaces.

[200] Pandey S., Shaikh F., Gupta A., Tripathi P., Yadav J.S. (2022). A recent update: Solid lipid nanoparticles for effective drug delivery. Adv. Pharm. Bull..

[201] Ghasemiyeh P., Mohammadi-Samani S. (2018). Solid lipid nanoparticles and nanostructured lipid carriers as novel drug delivery systems: Applications, advantages and disadvantages. Res. Pharm. Sci..

[202] Santos P., Almeida F. (2021). Exosome-based vaccines: History, current state, and clinical trials. Front. Immunol..

[203] Hazrati A., Soudi S., Malekpour K., Mahmoudi M., Rahimi A., Hashemi S.M., Varma R.S. (2022). Immune cells-derived exosomes function as a double-edged sword: Role in disease progression and their therapeutic applications. Biomark. Res..

[204] Dai J., Su Y., Zhong S., Cong L., Liu B., Yang J., Tao Y., He Z., Chen C., Jiang Y. (2020). Exosomes: Key players in cancer and potential therapeutic strategy. Signal Transduct. Target. Ther..

[205] Rezabakhsh A., Mahdipour M., Nourazarian A., Habibollahi P., Sokullu E., Avci Ç.B., Rahbarghazi R. (2022). Application of exosomes for the alleviation of COVID-19-related pathologies. Cell Biochem. Funct..

[206] Wallis J., Shenton D., Carlisle R. (2019). Novel approaches for the design, delivery and administration of vaccine technologies. Clin. Exp. Immunol..

[207] Glück R., Metcalfe I. (2002). New technology platforms in the development of vaccines for the future. Vaccine.

[208] O’Hagan D.T. (2007). MF59 is a safe and potent vaccine adjuvant that enhances protection against influenza virus infection. Expert Rev. Vaccines.

[209] Tang J.-l., Sun J., He Z.-G. (2007). Self-emulsifying drug delivery systems: Strategy for improving oral delivery of poorly soluble drugs. Curr. Drug Ther..

[210] Akagi T., Baba M., Akashi M. (2012). Biodegradable nanoparticles as vaccine adjuvants and delivery systems: Regulation of immune responses by nanoparticle-based vaccine. Polym. Nanomed..

[211] Liu B., Wu Z., Liu T., Qian R., Wu T., Liu Q., Shen A. (2018). Polymeric nanoparticles engineered as a vaccine adjuvant-delivery system. Immunization-Vaccine Adjuvant Delivery System and Strategies.

[212] Danhier F., Ansorena E., Silva J.M., Coco R., Le Breton A., Préat V. (2012). PLGA-based nanoparticles: An overview of biomedical applications. J. Control. Release.

[213] Reis C.P., Neufeld R.J., Veiga F., Ribeiro A.J. (2017). Preparation of drug-loaded polymeric nanoparticles. Nanomedicine in Cancer.

[214] Maji I., Mahajan S., Sriram A., Mehra N.K., Singh P.K. (2023). Polymeric nanomaterials: Fundamentals and therapeutic applications. Nanomaterial-Based Drug Delivery Systems: Therapeutic and Theranostic Applications.

[215] Cano A., Ettcheto M., Espina M., López-Machado A., Cajal Y., Rabanal F., Sánchez-López E., Camins A., García M.L., Souto E.B. (2020). State-of-the-art polymeric nanoparticles as promising therapeutic tools against human bacterial infections. J. Nanobiotechnology.

[216] Jin Z., Gao S., Cui X., Sun D., Zhao K. (2019). Adjuvants and delivery systems based on polymeric nanoparticles for mucosal vaccines. Int. J. Pharm..

[217] Elsabahy M., Wooley K.L. (2012). Design of polymeric nanoparticles for biomedical delivery applications. Chem. Soc. Rev..

[218] Cohn D., Stern T., González M.F., Epstein J. (2002). Biodegradable poly(ethylene oxide)/poly(ϵ-caprolactone) multiblock copolymers. J. Biomed. Mater. Res..

[219] Khodaverdi E., Tayarani-Najaran Z., Minbashi E., Alibolandi M., Hosseini J., Sepahi S., Kamali H., Hadizadeh F. (2019). Docetaxel-loaded mixed micelles and polymersomes composed of poly (caprolactone)-poly(ethylene glycol)(PEG-PCL) and poly (lactic acid)-poly (ethylene glycol)(PEG-PLA): Preparation and in-vitro characterization. Iran. J. Pharm. Res. IJPR.

[220] Tian J.-H., Patel N., Haupt R., Zhou H., Weston S., Hammond H., Logue J., Portnoff A.D., Norton J., Guebre-Xabier M. (2021). SARS-CoV-2 spike glycoprotein vaccine candidate NVX-CoV2373 immunogenicity in baboons and protection in mice. Nat. Commun..

[221] Bangaru S., Ozorowski G., Turner H.L., Antanasijevic A., Huang D., Wang X., Torres J.L., Diedrich J.K., Tian J.-H., Portnoff A.D. (2020). Structural analysis of full-length SARS-CoV-2 spike protein from an advanced vaccine candidate. Science.

[222] Bivas-Benita M., van Meijgaarden K.E., Franken K.L., Junginger H.E., Borchard G., Ottenhoff T.H., Geluk A. (2004). Pulmonary delivery of chitosan-DNA nanoparticles enhances the immunogenicity of a DNA vaccine encoding HLA-A* 0201-restricted T-cell epitopes of *Mycobacterium tuberculosis*. Vaccine.

[223] Mitchell M.J., Billingsley M.M., Haley R.M., Wechsler M.E., Peppas N.A., Langer R. (2021). Engineering precision nanoparticles for drug delivery. Nat. Rev. Drug Discov..

[224] Rezigue M. (2020). Lipid and Polymeric Nanoparticles: Drug Delivery Applications.

[225] Chenthamara D., Subramaniam S., Ramakrishnan S.G., Krishnaswamy S., Essa M.M., Lin F.-H., Qoronfleh M.W. (2019). Therapeutic efficacy of nanoparticles and routes of administration. Biomater. Res..

[226] Elzoghby A., M Abd-Elwakil M., Abd-Elsalam K., T Elsayed M., Hashem Y., Mohamed O. (2016). Natural polymeric nanoparticles for brain-targeting: Implications on drug and gene delivery. Curr. Pharm. Des..

[227] Sun Z., Zhao H., Ma L., Shi Y., Ji M., Sun X., Ma D., Zhou W., Huang T., Zhang D. (2024). The quest for nanoparticle-powered vaccines in cancer immunotherapy. J. Nanobiotechnol..

[228] Dunkle L.M., Kotloff K.L., Gay C.L., Áñez G., Adelglass J.M., Barrat Hernández A.Q., Harper W.L., Duncanson D.M., McArthur M.A., Florescu D.F. (2022). Efficacy and safety of NVX-CoV2373 in adults in the United States and Mexico. N. Engl. J. Med..

[229] Vasudevan S.S., Kandrikar T.Y., Sayyed A.A., Sarker P., Nasir N.S., Venugopalan S., Mariajohn R., Chavda V.P., Gondaliya P. (2024). Nanoparticle-based vaccines and future vaccine technologies. Advanced Vaccination Technologies for Infectious and Chronic Diseases.

[230] Cable J., Graham B.S., Koup R.A., Seder R.A., Karikó K., Pardi N., Barouch D.H., Sharma B., Rauch S., Nachbagauer R. (2023). Progress in vaccine development for infectious diseases—A Keystone Symposia report. Ann. N. Y. Acad. Sci..

[231] Kasturi S.P., Skountzou I., Albrecht R.A., Koutsonanos D., Hua T., Nakaya H.I., Ravindran R., Stewart S., Alam M., Kwissa M. (2011). Programming the magnitude and persistence of antibody responses with innate immunity. Nature.

[232] Hess K.L., Medintz I.L., Jewell C.M. (2019). Designing inorganic nanomaterials for vaccines and immunotherapies. Nano Today.

[233] Joseph T.M., Kar Mahapatra D., Esmaeili A., Piszczyk Ł., Hasanin M.S., Kattali M., Haponiuk J., Thomas S. (2023). Nanoparticles: Taking a unique position in medicine. Nanomaterials.

[234] Bansal S.A., Kumar V., Karimi J., Singh A.P., Kumar S. (2020). Role of gold nanoparticles in advanced biomedical applications. Nanoscale Adv..

[235] Amendola V., Pilot R., Frasconi M., Maragò O.M., Iatì M.A. (2017). Surface plasmon resonance in gold nanoparticles: A review. J. Phys. Condens. Matter.

[236] Gnanaraj M., Sisubalan N., Jebastin T., Vijayan A., Muneeshwaran T., Manikandan R. (2024). Gold Nanoparticles as Antibacterial and Antiviral Agents: Biomedical Applications and Theranostic Potential. Nanoparticles in Modern Antimicrobial and Antiviral Applications.

[237] Chaudhary K., Masram D.T. (2020). Biological activities of nanoparticles and mechanism of action. Model Organisms to Study Biological Activities and Toxicity of Nanoparticles.

[238] Chernykh I., Kopanitsa M., Shchul’kin A., Yakusheva E., Frolova M. (2021). Gold nanoparticles as potential antitumor agents. Pharm. Chem. J..

[239] He J.-s., Liu S.-j., Zhang Y.-r., Chu X.-d., Lin Z.-b., Zhao Z., Qiu S.-h., Guo Y.-g., Ding H., Pan Y.-l. (2021). The application of and strategy for gold nanoparticles in cancer immunotherapy. Front. Pharmacol..

[240] Farfán-Castro S., García-Soto M.J., Aguilar-Aguilar A., González-Ortega O., Rosales-Mendoza S. (2024). Application of gold nanoparticles in vaccine development. Gold Nanoparticles for Drug Delivery.

[241] Xu L., Wang X., Wang W., Sun M., Choi W.J., Kim J.-Y., Hao C., Li S., Qu A., Lu M. (2022). Enantiomer-dependent immunological response to chiral nanoparticles. Nature.

[242] Tapia D., Sanchez-Villamil J.I., Torres A.G. (2020). Multicomponent gold nano-glycoconjugate as a highly immunogenic and protective platform against *Burkholderia mallei*. npj Vaccines.

[243] Gao L., Song Y., Zhong J., Lin X., Zhou S.-F., Zhan G. (2022). Biocompatible 2D Cu-TCPP nanosheets derived from Cu_2_O nanocubes as multifunctional nanoplatforms for combined anticancer therapy. ACS Biomater. Sci. Eng..

[244] Sztandera K., Gorzkiewicz M., Klajnert-Maculewicz B. (2018). Gold nanoparticles in cancer treatment. Mol. Pharm..

[245] Vines J.B., Yoon J.-H., Ryu N.-E., Lim D.-J., Park H. (2019). Gold nanoparticles for photothermal cancer therapy. Front. Chem..

[246] Singh P., Mijakovic I. (2021). Advances in gold nanoparticle technology as a tool for diagnostics and treatment of cancer. Expert Rev. Mol. Diagn..

[247] Nandanwar R., Singh P., Haque F.Z. (2013). Synthesis and properties of silica nanoparticles by sol-gel method for the application in green chemistry. Mater. Sci. Res. India.

[248] Bharti C., Nagaich U., Pal A.K., Gulati N. (2015). Mesoporous silica nanoparticles in target drug delivery system: A review. Int. J. Pharm. Investig..

[249] Tang F., Li L., Chen D. (2012). Mesoporous silica nanoparticles: Synthesis, biocompatibility and drug delivery. Adv. Mater..

[250] Scheiblhofer S., Machado Y., Feinle A., Thalhamer J., Hüsing N., Weiss R. (2016). Potential of nanoparticles for allergen-specific immunotherapy–use of silica nanoparticles as vaccination platform. Expert Opin. Drug Deliv..

[251] Seré S., Vounckx U., Seo J.W., Lenaerts I., Van Gool S., Locquet J.-P. (2020). Proof of concept study: Mesoporous silica nanoparticles, from synthesis to active specific immunotherapy. Front. Nanotechnol..

[252] Liu J.Y., Sayes C.M. (2022). A toxicological profile of silica nanoparticles. Toxicol. Res..

[253] Pallavi P., Harini K., Alshehri S., Ghoneim M.M., Alshlowi A., Gowtham P., Girigoswami K., Shakeel F., Girigoswami A. (2022). From synthetic route of silica nanoparticles to theranostic applications. Processes.

[254] Xiao D., Qi H., Teng Y., Pierre D., Kutoka P.T., Liu D. (2021). Advances and challenges of fluorescent nanomaterials for synthesis and biomedical applications. Nanoscale Res. Lett..

[255] Nooraei S., Bahrulolum H., Hoseini Z.S., Katalani C., Hajizade A., Easton A.J., Ahmadian G. (2021). Virus-like particles: Preparation, immunogenicity and their roles as nanovaccines and drug nanocarriers. J. Nanobiotechnol..

[256] Liu X., Liu Y., Yang X., Lu X., Xu X.-N., Zhang J., Chen R. (2023). Potentiating the Immune Responses of HBsAg-VLP Vaccine Using a Polyphosphoester-Based Cationic Polymer Adjuvant. ACS Appl. Mater. Interfaces.

[257] Gupta R., Arora K., Roy S.S., Joseph A., Rastogi R., Arora N.M., Kundu P.K. (2023). Platforms, advances, and technical challenges in virus-like particles-based vaccines. Front. Immunol..

[258] Chu K.-B., Quan F.-S. (2021). Virus-Like Particle Vaccines Against Respiratory Viruses and Protozoan Parasites.

[259] Patil S. (2021). A review of virus-like particle-based SARS-CoV-2 vaccines in clinical trial phases. Iran. J. Pharm. Res. IJPR.

[260] Aboshi M., Matsuda K., Kawakami D., Kono K., Kazami Y., Sekida T., Komori M., Morey A.L., Suga S., Smith J.F. (2024). Safety and immunogenicity of VLPCOV-02, a SARS-CoV-2 self-amplifying RNA vaccine with a modified base, 5-methylcytosine. iScience.

[261] Polack F.P., Thomas S.J., Kitchin N., Absalon J., Gurtman A., Lockhart S., Perez J.L., Pérez Marc G., Moreira E.D., Zerbini C. (2020). Safety and efficacy of the BNT162b2 mRNA COVID-19 vaccine. N. Engl. J. Med..

[262] Vandoolaeghe P., Schuerman L. (2016). The RTS, S/AS01 malaria vaccine in children 5 to 17 months of age at first vaccination. Expert Rev. Vaccines.

[263] Syomin B., Ilyin Y. (2019). Virus-like particles as an instrument of vaccine production. Mol. Biol..

[264] Erdmann N.B., Williams W.B., Walsh S.R., Grunenberg N., Edlefsen P.T., Goepfert P.A., Cain D.W., Cohen K.W., Maenza J., Mayer K.H. (2024). A HIV-1 Gp41 peptide-liposome vaccine elicits neutralizing epitope-targeted antibody responses in healthy individuals. medRxiv.

[265] Zeigler D.F., Gage E., Roque R., Clegg C.H. (2019). Epitope targeting with self-assembled peptide vaccines. NPJ Vaccines.

[266] Walls A.C., Fiala B., Schäfer A., Wrenn S., Pham M.N., Murphy M., Longping V.T., Shehata L., O’Connor M.A., Chen C. (2020). Elicitation of potent neutralizing antibody responses by designed protein nanoparticle vaccines for SARS-CoV-2. Cell.

[267] Yang J., Wang W., Chen Z., Lu S., Yang F., Bi Z., Bao L., Mo F., Li X., Huang Y. (2020). A vaccine targeting the RBD of the S protein of SARS-CoV-2 induces protective immunity. Nature.

[268] López-Sagaseta J., Malito E., Rappuoli R., Bottomley M.J. (2016). Self-assembling protein nanoparticles in the design of vaccines. Comput. Struct. Biotechnol. J..

[269] Zhao L., Seth A., Wibowo N., Zhao C.-X., Mitter N., Yu C., Middelberg A.P. (2014). Nanoparticle vaccines. Vaccine.

[270] Bagheri-Josheghani S., Bakhshi B., Najar-peerayeh S. (2022). The influence of nanoparticle on vaccine responses against bacterial infection. J. Nanotechnol..

[271] Chang H.C., Zou Z.Z., Wang Q.H., Li J., Jin H., Yin Q.X., Xing D. (2020). Targeting and specific activation of antigen--presenting cells by endogenous antigen-loaded nanoparticles elicits tumor-specific immunity. Adv. Sci..

[272] Gamucci O., Bertero A., Gagliardi M., Bardi G. (2014). Biomedical nanoparticles: Overview of their surface immune-compatibility. Coatings.

[273] Zaman M., Good M.F., Toth I. (2013). Nanovaccines and their mode of action. Methods.

[274] Lin Y., Chen X., Wang K., Liang L., Zhang H. (2024). An Overview of Nanoparticle-Based Delivery Platforms for mRNA Vaccines for Treating Cancer. Vaccines.

[275] Oberli M.A., Reichmuth A.M., Dorkin J.R., Mitchell M.J., Fenton O.S., Jaklenec A., Anderson D.G., Langer R., Blankschtein D. (2017). Lipid nanoparticle assisted mRNA delivery for potent cancer immunotherapy. Nano Lett..

[276] Pardi N., Hogan M.J., Porter F.W., Weissman D. (2018). mRNA vaccines—A new era in vaccinology. Nat. Rev. Drug Discov..

[277] Han Y., Renu S., Patil V., Schrock J., Feliciano-Ruiz N., Selvaraj R., Renukaradhya G.J. (2020). Mannose-modified chitosan-nanoparticle-based *Salmonella* subunit oralvaccine-induced immune response and efficacy in a challenge trial in broilers. Vaccines.

[278] Dolatyabi S., Renu S., Schrock J., Renukaradhya G.J. (2024). Chitosan-nanoparticle-based oral *Salmonella* enteritidis subunit vaccine elicits cross-protection against *Salmonella* typhimurium in broilers. Poult. Sci..

[279] Tapia D. Evaluation of a Gold Nanoparticle Platform as Highly Immunogenic and Protective Therapy Against *Burkholderia Mallei*, *B. pseudomallei*, and Enterohemorrhagic *Escherichia coli* O157: H7. Ph.D. Published ETD Collection 2022. https://hdl.handle.net/2152.3/11436.

[280] Zagaglia C., Ammendolia M.G., Maurizi L., Nicoletti M., Longhi C. (2022). Urinary tract infections caused by uropathogenic *Escherichia coli* strains—New strategies for an old pathogen. Microorganisms.

[281] Wang X., Gao X., Wang L., Lin J., Liu Y. (2023). Advances of Nanotechnology Toward Vaccine Development Against Animal Infectious Diseases. Adv. Funct. Mater..

[282] Mat Rani N.N.I., Alzubaidi Z.M., Butt A.M., Mohammad Faizal N.D.F., Sekar M., Azhari H., Mohd Amin M.C.I. (2022). Outer membrane vesicles as biomimetic vaccine carriers against infections and cancers. Wiley Interdiscip. Rev. Nanomed. Nanobiotechnol..

[283] Mawad A., Helmy Y.A., Shalkami A.-G., Kathayat D., Rajashekara G.E. (2018). *coli* Nissle microencapsulation in alginate-chitosan nanoparticles and its effect on *Campylobacter jejuni* in vitro. Appl. Microbiol. Biotechnol..

[284] Helmy Yosra A., Closs G., Jung K., Kathayat D., Vlasova A., Rajashekara G. (2022). Effect of Probiotic *E. coli* Nissle 1917 Supplementation on the Growth Performance, Immune Responses, Intestinal Morphology, and Gut Microbes of Campylobacter jejuni Infected Chickens. Infect. Immun..

[285] Oladejo M., Paterson Y., Wood L.M. (2021). Clinical experience and recent advances in the development of listeria-based tumor immunotherapies. Front. Immunol..

[286] Datta M., Rajeev A., Chattopadhyay I. (2023). Application of antimicrobial peptides as next-generation therapeutics in the biomedical world. Biotechnol. Genet. Eng. Rev..

[287] Yao J., Chen Y., Zhang L., Cheng Y., Chen Z., Zhang Y., Zheng X., Lv Y., Wang S., Li Z. (2024). pH-responsive CuS/DSF/EL/PVP nanoplatform alleviates inflammatory bowel disease in mice via regulating gut immunity and microbiota. Acta Biomater..

[288] Patra J.K., Das G., Fraceto L.F., Campos E.V.R., Rodriguez-Torres M.d.P., Acosta-Torres L.S., Diaz-Torres L.A., Grillo R., Swamy M.K., Sharma S. (2018). Nano based drug delivery systems: Recent developments and future prospects. J. Nanobiotechnol..

[289] Reddy P.R.K., Yasaswini D., Reddy P.P.R., Kumar D.S., Elghandour M.M., Salem A. (2023). Nanotechnology in Veterinary Sector: Current Applications, Limitations and Future Perspective. Handbook of Green and Sustainable Nanotechnology: Fundamentals, Developments and Applications.

[290] Celis-Giraldo C.T., López-Abán J., Muro A., Patarroyo M.A., Manzano-Román R. (2021). Nanovaccines against animal pathogens: The latest findings. Vaccines.

[291] Renu S., Han Y., Dhakal S., Lakshmanappa Y.S., Ghimire S., Feliciano-Ruiz N., Senapati S., Narasimhan B., Selvaraj R., Renukaradhya G.J. (2020). Chitosan-adjuvanted *Salmonella* subunit nanoparticle vaccine for poultry delivered through drinking water and feed. Carbohydr. Polym..

[292] Wang X., Dai Y., Zhao S., Tang J., Li H., Xing Y., Qu G., Li X., Dai J., Zhu Y. (2014). PAMAM-Lys, a novel vaccine delivery vector, enhances the protective effects of the SjC23 DNA vaccine against *Schistosoma japonicum* infection. PLoS ONE.

[293] Coleman C.M., Liu Y.V., Mu H., Taylor J.K., Massare M., Flyer D.C., Glenn G.M., Smith G.E., Frieman M.B. (2014). Purified coronavirus spike protein nanoparticles induce coronavirus neutralizing antibodies in mice. Vaccine.

[294] Al-Halifa S., Gauthier L., Arpin D., Bourgault S., Archambault D. (2019). Nanoparticle-based vaccines against respiratory viruses. Front. Immunol..

[295] Zhu D., Long Q., Xu Y., Xing J. (2019). Evaluating nanoparticles in preclinical research using microfluidic systems. Micromachines.

[296] Desai N., Chavda V., Singh T.R.R., Thorat N.D., Vora L.K. (2024). Cancer nanovaccines: Nanomaterials and clinical perspectives. Small.

[297] Zhao G., Jiang Y., Ma P., Wang S., Nie G., Li N. (2023). Membrane-based cancer nanovaccines: The time is now. QJM Int. J. Med..

[298] Sharma H., Thakur S. (2024). 2 Membrane-Based Nano. Nanoparticles in Cancer Therapy: Innovations and Clinical Applications.

[299] Liu Y., Cheng W., Xin H., Liu R., Wang Q., Cai W., Peng X., Yang F., Xin H. (2023). Nanoparticles advanced from preclinical studies to clinical trials for lung cancer therapy. Cancer Nanotechnol..

[300] Al-Thani A.N., Jan A.G., Abbas M., Geetha M., Sadasivuni K.K. (2024). Nanoparticles in cancer theragnostic and drug delivery: A comprehensive review. Life Sci..

[301] Sun L., Liu H., Ye Y., Lei Y., Islam R., Tan S., Tong R., Miao Y.-B., Cai L. (2023). Smart nanoparticles for cancer therapy. Signal Transduct. Target. Ther..

[302] Selvaraj J., Rajendran V., Ramalingam B. (2021). Nanovaccine: A Modern Approach to Vaccinology. Nanotechnol. Med..

[303] Sun M., Pratama A.C., Qiu H., Liu Z., He F. (2024). Toward innovative veterinary nanoparticle vaccines. Anim. Dis..

[304] Geng Q., Tai W., Baxter V.K., Shi J., Wan Y., Zhang X., Montgomery S.A., Taft-Benz S.A., Anderson E.J., Knight A.C. (2021). Novel virus-like nanoparticle vaccine effectively protects animal model from SARS-CoV-2 infection. PLoS Pathog..

[305] Duvall M.N., Knight K. (2012). FDA Regulation of Nanotechnology.

[306] Csóka I., Ismail R., Jójárt-Laczkovich O., Pallagi E. (2021). Regulatory considerations, challenges and risk-based approach in nanomedicine development. Curr. Med. Chem..

[307] Paliwal R., Kumar P., Chaurasiya A., Kenwat R., Katke S., Paliwal S.R. (2022). Development of nanomedicines and nano-similars: Recent advances in regulatory landscape. Curr. Pharm. Des..

[308] Souto E.B., Blanco-Llamero C., Krambeck K., Kiran N.S., Yashaswini C., Postwala H., Severino P., Priefer R., Prajapati B.G., Maheshwari R. (2024). Regulatory Insights into Nanomedicine and Gene Vaccine Innovation: Safety Assessment, Challenges, and Regulatory Perspectives. Acta Biomater..

[309] Gogoi N.R., Bezbaruah R., Patel V., Satasia R., Bhattacharjee B., Mazumder B. (2024). Regulatory Pathways for Nanocarrier Vaccine. Nanocarrier Vaccines: Biopharmaceutics-Based Fast Track Development.

[310] Florindo H., Lopes J., Silva L., Corvo M., Martins M., Gaspar R. (2017). Regulatory development of nanotechnology-based vaccines. Micro and Nanotechnology in Vaccine Development.

[311] Araste F., Bakker A.D., Zandieh-Doulabi B. (2023). Potential and risks of nanotechnology applications in COVID-19-related strategies for pandemic control. J. Nanoparticle Res..

[312] World Health Organization (2009). State of the World’s Vaccines and Immunization.

[313] Fernandes C., Jathar M., Sawant B.K.S., Warde T. (2023). Scale-up of nanoparticle manufacturing process. Pharmaceutical Process Engineering and Scale-Up Principles.

[314] Liu X., Meng H. (2021). Consideration for the scale-up manufacture of nanotherapeutics—A critical step for technology transfer. View.

[315] Borrajo M.L., Lou G., Anthiya S., Lapuhs P., Álvarez D.M., Tobío A., Loza M.I., Vidal A., Alonso M.J. (2024). Nanoemulsions and nanocapsules as carriers for the development of intranasal mRNA vaccines. Drug Delivery and Translational Research.

[316] Hu C., Bai Y., Liu J., Wang Y., He Q., Zhang X., Cheng F., Xu M., Mao Q., Liang Z. (2024). Research progress on the quality control of mRNA vaccines. Expert Rev. Vaccines.

[317] Whitley J., Zwolinski C., Denis C., Maughan M., Hayles L., Clarke D., Snare M., Liao H., Chiou S., Marmura T. (2022). Development of mRNA manufacturing for vaccines and therapeutics: mRNA platform requirements and development of a scalable production process to support early phase clinical trials. Transl. Res..

[318] Rosa S.S., Prazeres D.M., Azevedo A.M., Marques M.P. (2021). mRNA vaccines manufacturing: Challenges and bottlenecks. Vaccine.

[319] Guerrini G., Magrì D., Gioria S., Medaglini D., Calzolai L. (2022). Characterization of nanoparticles-based vaccines for COVID-19. Nat. Nanotechnol..

[320] Lee J.H., Yeo Y. (2015). Controlled drug release from pharmaceutical nanocarriers. Chem. Eng. Sci..

[321] Svenson S., Tomalia D.A. (2012). Dendrimers in biomedical applications—Reflections on the field. Adv. Drug Deliv. Rev..

[322] Jain K., Kesharwani P., Gupta U., Jain N. (2010). Dendrimer toxicity: Let’s meet the challenge. Int. J. Pharm..

[323] George E., Goswami A., Lodhiya T., Padwal P., Iyer S., Gauttam I., Sethi L., Jeyasankar S., Sharma P.R., Dravid A.A. (2022). Immunomodulatory effect of mycobacterial outer membrane vesicles coated nanoparticles. Biomater. Adv..

[324] Wang S., Gao J., Wang Z. (2019). Outer membrane vesicles for vaccination and targeted drug delivery. Wiley Interdiscip. Rev. Nanomed. Nanobiotechnol..

[325] Zheng Y., Zhao Y., Bai M., Gu H., Li X. (2022). Metal–organic frameworks as a therapeutic strategy for lung diseases. J. Mater. Chem. B.

[326] Tousian B., Khosravi A.R., Ghasemi M.H., Kadkhodaie M. (2024). Biomimetic Functionalized Metal Organic Frameworks as Multifunctional Agents: Paving the Way for Cancer Vaccine Advances. Mater. Today Bio.

[327] Manimaran V., Nivetha R., Tamilanban T., Narayanan J., Vetriselvan S., Fuloria N.K., Chinni S.V., Sekar M., Fuloria S., Wong L.S. (2023). Nanogels as novel drug nanocarriers for CNS drug delivery. Front. Mol. Biosci..

[328] Nakahashi-Ouchida R., Yuki Y., Kiyono H. (2017). Development of a nanogel-based nasal vaccine as a novel antigen delivery system. Expert Rev. Vaccines.

[329] Kabanov A.V., Vinogradov S.V. (2009). Nanogels as pharmaceutical carriers: Finite networks of infinite capabilities. Angew. Chem. Int. Ed..

[330] Shinde V., Fries L., Wu Y., Agrawal S., Cho I., Thomas D.N., Spindler M., Lindner E., Hahn T., Plested J. (2018). Improved titers against influenza drift variants with a nanoparticle vaccine. N. Engl. J. Med..

[331] Brazzoli M., Piccioli D., Marchetti F. (2023). Challenges in development of vaccines directed toward antimicrobial resistant bacterial species. Hum. Vaccines Immunother..

[332] Weber J.S., Carlino M.S., Khattak A., Meniawy T., Ansstas G., Taylor M.H., Kim K.B., McKean M., Long G.V., Sullivan R.J. (2024). Individualised neoantigen therapy mRNA-4157 (V940) plus pembrolizumab versus pembrolizumab monotherapy in resected melanoma (KEYNOTE-942): A randomised, phase 2b study. Lancet.

[333] Saied A.A. (2022). mRNA vaccines and clinical research in Africa-From hope to reality. Int. J. Surg..

[334] Sekiya T., Ohno M., Nomura N., Handabile C., Shingai M., Jackson D.C., Brown L.E., Kida H. (2021). Selecting and using the appropriate influenza vaccine for each individual. Viruses.

[335] Chu Y., Liu Q., Wei J., Liu B. (2018). Personalized cancer neoantigen vaccines come of age. Theranostics.

[336] Zhao W., Wu W., Xu X. (2007). Oral vaccination with liposome-encapsulated recombinant fusion peptide of urease B epitope and cholera toxin B subunit affords prophylactic and therapeutic effects against *H. pylori* infection in BALB/c mice. Vaccine.

[337] Kim B., Bowersock T., Griebel P., Kidane A., Babiuk L., Sanchez M., Attah-Poku S., Kaushik R., Mutwiri G. (2002). Mucosal immune responses following oral immunization with rotavirus antigens encapsulated in alginate microspheres. J. Control. Release.

[338] Riccardi D., Baldino L., Reverchon E. (2024). Liposomes, transfersomes and niosomes: Production methods and their applications in the vaccinal field. J. Transl. Med..

[339] Saggese A., Baccigalupi L., Donadio G., Ricca E., Isticato R. (2023). The bacterial spore as a mucosal vaccine delivery system. Int. J. Mol. Sci..

[340] Abouelela M.E., Helmy Y.A. (2024). Next-Generation Probiotics as Novel Therapeutics for Improving Human Health: Current Trends and Future Perspectives. Microorganisms.

[341] Chin J.S., Chooi W.H., Wang H., Ong W., Leong K.W., Chew S.Y. (2019). Scaffold-mediated non-viral delivery platform for CRISPR/Cas9-based genome editing. Acta Biomater..

[342] Hii A.R.K., Qi X., Wu Z. (2024). Advanced strategies for CRISPR/Cas9 delivery and applications in gene editing, therapy, and cancer detection using nanoparticles and nanocarriers. J. Mater. Chem. B.

[343] Li J., Li J., Peng Y., Du Y., Yang Z., Qi X. (2023). Dendritic cell derived exosomes loaded neoantigens for personalized cancer immunotherapies. J. Control. Release.

[344] Ott P.A., Hu Z., Keskin D.B., Shukla S.A., Sun J., Bozym D.J., Zhang W., Luoma A., Giobbie-Hurder A., Peter L. (2017). An immunogenic personal neoantigen vaccine for patients with melanoma. Nature.

[345] Yao R., Xie C., Xia X. (2024). Recent progress in mRNA cancer vaccines. Hum. Vaccines Immunother..

[346] Mi Y., Smith C.C., Serody J.S., Vincent B.G., Wang A.Z., Hyun H. (2020). Neoantigen nanovaccine improves personalized cancer immunotherapy. Cancer Res..

[347] Feng R., Yu F., Xu J., Hu X. (2021). Knowledge gaps in immune response and immunotherapy involving nanomaterials: Databases and artificial intelligence for material design. Biomaterials.

[348] Zohuri B., Behgounia F. (2023). Application of artificial intelligence driving nano-based drug delivery system. A Handbook of Artificial Intelligence in Drug Delivery.

[349] Hasanzadeh A., Hamblin M.R., Kiani J., Noori H., Hardie J.M., Karimi M., Shafiee H. (2022). Could artificial intelligence revolutionize the development of nanovectors for gene therapy and mRNA vaccines?. Nano Today.

[350] Hamilton S., Kingston B.R. (2024). Applying artificial intelligence and computational modeling to nanomedicine. Curr. Opin. Biotechnol..

[351] Shi Y., Chen L., Zhu M., Zhao Y. (2022). The Future of Nanomedicine. Nanomedicine.

